# Decomposing difference in the kidney cancer burden measures between 1990 and 2019 based on the global burden of disease study

**DOI:** 10.1038/s41598-024-61300-2

**Published:** 2024-05-06

**Authors:** Erfan Ayubi, Fatemeh Shahbazi, Salman Khazaei

**Affiliations:** 1 Cancer Research Center, Hamadan University of Medical Sciences, Hamadan, Iran; 2 Department of Epidemiology, School of Health, Hamadan University of Medical Sciences, Hamadan, Iran; 3 Research Center for Health Sciences, Hamadan University of Medical Sciences, Hamadan, Iran

**Keywords:** Kidney cancer, Risk factor, Decomposition analysis, Global burden study, Cancer, Risk factors

## Abstract

The kidney cancer (KC) burden measures have changed dramatically in recent years due to changes in exposure to the determinants over time. We aimed to decompose the difference in the KC burden measures between 1990 and 2019. This ecological study included data on the KC burden measures as well as socio-demographic index (SDI), behavioral, dietary, and metabolic risk factors from the global burden of disease study. Non-linear multivariate decomposition analysis was applied to decompose the difference in the burden of KC. Globally, ASIR, ASMR, and ASDR of KC increased from 2.88 to 4.37, from 1.70 to 2.16, and from 46.13 to 54.96 per 100,000 people between 1990 and 2019, respectively. The global burden of KC was more concentrated in developed countries. From 1990 to 2019, the burden of KC has increased the most in Eastern European countries. More than 70% of the difference in the KC burden measures between 1990 and 2019 was due to changes in exposure to the risk factors over time. The SDI, high body mass index (BMI), and alcohol use had the greatest contribution to the difference in the KC burden measures. Changes in characteristics over time, including SDI, high BMI, and alcohol consumption, appear to be important in the evolving landscape of KC worldwide. This finding may help policymakers design policies and implement prevention programs to control and manage KC.

## Introduction

Globally, kidney cancer (KC) is a most prevalent urinary tract cancer. The incident cases of KC increased from 207.31 thousand in 1990 to 393.04 thousand in 2017. Additionally, KC deaths increased from 68.14 thousand in 1990 to 138.53 in 2017^1,2^. The KC is varied by geography, sex, and age. The developed countries demonstrate the highest incidence and mortality rates, although the trend is expected to level off and decrease in these countries over the next decade^3^. However, the incidence and mortality of KC are currently low in developing and less developed countries and are expected to increase in these countries in the coming years^3^. Most cases of KC occur in men and between the ages of 40 and 65^2–4^.

The etiology of KC is multifactorial. Hypertension, obesity, and smoking are the well-known risk factors^5–7^. The prevalence rate of chronic kidney diseases (CKD) among KC patients can reach to 72%^8^. Alcohol consumption, diabetes mellitus, and occupational exposure to trichloroethylene are the debated and suspected risk factors^5–7^. The environmental risk factors mentioned above may independently or through a synergistic interaction increase the risk of KC incidence and mortality. Previous studies have considered the role of socioeconomic status on the KC burden and variation in KC risk factors^9–11^.

The changes in exposure to risk factors affect the burden of KC in recent years^12^. Assessing differences in health outcomes and risk factors influencing them over time and, also comparing statistics at different time points can be helpful for planning to reduce the burden of KC. It is critical to quantify differences in health outcomes and their determinants over time and across different groups^13^. Hence, it may be good to know how much of the difference in the burden of KC between two periods (e.g., 1990 and 2019) can be explained by the distribution of risk factors, and also which risk factors have the most contribution to the observed difference. Given the type and scale of the health outcome, various inequality statistical methods exist to quantify temporal-group difference and also the main determinants of temporal-group differences^14^.

The Global Burden of Diseases (GBD) study provides reliable and accurate estimates of burden of diseases and risk factors for different regions of the world^15^. Although there is robust evidence of global trends in KC and its risk factors from GBD studies^2–4^, however, it is useful that it provided new insights into the risk factors influencing changes in KC burden over time using different statistical methods. Therefore, this study aimed to partition the difference in the burden of KC between 1990 and 2019 into major risk factors using multivariate decomposition analysis.

## Methods

### Overview of the GBD study and dataset

The GBD Study provides the detailed information regarding 367 causes of death and disability and 87 risk factors for 204 countries and territories^15^. In the present study, we analyzed GBD data on age-standardized incidence rate (ASIR), age-standardized mortality rate (ASMR), and age-standardized DALY rate (ASDR) of KC and its risk factors per 100,000 people. Information regarding behavioral, dietary and metabolic risk factors of KC such as high body mass index (BMI), high systolic blood pressure (SBP), smoking, alcohol use, diet low in fruit, diet low in vegetable, lower physical activity and occupation exposure to trichloroethylene was extracted from GBD summary exposure value (SEV) data. The SEV is an adjusted prevalence of exposure that ranged from 0 to 100%. The value of 0 indicates no exposure in a population and 100% represents maximum exposure in an entire population. We also extracted information regarding type 2 diabetes mellitus (T2DM) and CKD and socio-demographic index (SDI). The GBD developed the SDI as an indicator of the social development of countries and territories. The SDI consists of a combination of three variables: income, education and fertility and ranges from 0 to 1. Higher values of SDI indicate greater development. All data for this study is publicly available at https://vizhub.healthdata.org/gbd-results/.

### Non-linear multivariate decomposition analysis

We performed a non-linear multivariate decomposition analysis using Power et al. proposed technique^16^.

Consider a Poisson model where the outcome variable (e.g., count or rate) is a linear function of the explanatory variables and the coefficients:

$${\text{Y}} = {\text{F}}\left( {{\text{X}}\beta } \right)$$

where Y is outcome vector, X is vector of the explanatory variables, $\beta$ is vector of coefficients and F(.) is function mapping a linear combination of X (Xβ) to Y.

In a common-sense epidemiology, the values of ASIR, ASMR and ASDR for 1990 and 2019 can be considered as the total number of incidence, deaths and DALY (Y) divided by the total population (R). Expected rate can expressed in 2019 as

$$\begin{aligned} {\text{Expected}}\;{\text{rate}}, \quad \overline{\lambda }_{2019} = & \frac{{\sum Y_{2019} }}{{\sum R_{2019} }} = \frac{{\overline{Y}_{2019} }}{{\overline{R}_{2019} }} = \frac{{\sum F\left( {X_{2019} \beta_{2019} + logR_{2019} } \right)}}{{\sum R_{2019} }} \\ & = \frac{{\overline{{F\left( {X_{2019} \beta_{2019} + logR_{2019} } \right)}} }}{{\overline{R}_{2019} }} \\ \end{aligned}$$

Then, the mean difference in ASIR, ASMR and ASDR between 1990 and 2019 with offset term can be decomposed as;

$$\begin{gathered} \frac{{\overline{Y}_{2019} }}{{\overline{R}_{2019} }} - \frac{{\overline{Y}_{1990} }}{{\overline{R}_{1990} }} = \frac{{\overline{{F\left( {X_{2019} \beta_{2019} + logR_{2019} } \right)}} }}{{\overline{R}_{2019} }} - \frac{{\overline{{F\left( {X_{1990} \beta_{1990} + logR_{1990} } \right)}} }}{{\overline{R}_{1990} }} \hfill \\ = \underbrace {{\left\{ {\frac{{\overline{{F(X_{2019} \beta_{2019} + logR_{2019} }} )}}{{\overline{R}_{2019} }} - \frac{{\overline{{F\left( {X_{1990} \beta_{2019} + logR_{1990} } \right)}} }}{{\overline{R}_{1990} }}} \right\}}}_{{ = \left\{ {{\text{Differences}}\;{\text{in}}\;{\text{Endowment}}\;{\text{part}}} \right\} + }} + \underbrace {{\left\{ {\frac{{\overline{{F\left( {X_{1990} \beta_{2019} + logR_{1990} } \right)}} }}{{\overline{R}_{1990} }} - \frac{{\overline{{F\left( {X_{1990} \beta_{1990} + logR_{1990} } \right)}} }}{{\overline{R}_{1990} }}} \right\}}}_{{\left\{ {{\text{Differences}}\;{\text{in}}\;{\text{Coefficients}}\;{\text{part}}} \right\}}} \hfill \\ \end{gathered}$$

Here, we have used the model without offset term and then, log R = 0 and R = $\overline{R }$=1.

The first part is the endowment effect (E) and refers to differences in characteristics, known as the "explained part". The second part is the coefficient effect (C) and refers to the difference in coefficients, called the "unexplained part". In the detailed decomposition, the contribution of the exploratory variables on the E and C components can be evaluated if the distribution and coefficients of the variables of one group are successively replaced by the variables of the other group, while the other variables of the model is constant.

Unlike the linear model, the nonlinear multivariate decomposition method effectively handles the path dependence and identification problem^16^. Path dependence occurs when the order of covariates included in the decomposition can affect the results, and the identification problem occurs when dummy and nominal variables are introduced into the decomposition and are needed to select the omitted (baseline) level^14^. Several solutions (e.g., randomization of variables ordering across replications of the decomposition) have been introduced to solve the common problems of decomposition analysis^17,18^. A simpler and more efficient solution is to consider the weight component of E and C, which is independent of the order of entering the variables into decomposition analysis^19–21^.

The weight of the exploratory variable in the linearization of E and C is as follow;

$$W_{{\Delta_{{X_{k} }} }} = \beta_{{2019_{k} }} \left( {\overline{X}_{{2019_{k} }} - \overline{X}_{{1990_{k} }} } \right)/\mathop \sum \limits_{k = 1}^{K} \beta_{{2019_{k} }} \left( {\overline{X}_{{2019_{k} }} - \overline{X}_{{1990_{k} }} } \right)$$

$$W_{{\Delta_{{\beta_{k} }} }} = \overline{X}_{{2019_{k} }} \left( {\beta_{{2019_{k} }} - \beta_{{1990_{k} }} } \right)/\mathop \sum \limits_{k = 1}^{K} \overline{X}_{{2019_{k} }} \left( {\beta_{{2019_{k} }} - \beta_{{1990_{k} }} } \right)$$

where

$$\mathop \sum \limits_{k} W_{{\Delta_{{X_{k} }} }} = \mathop \sum \limits_{k} W_{{\Delta_{{\beta_{k} }} }} = 1.0$$

Then, difference decomposition can also be expressed as,

$$\frac{{\overline{Y}_{2019} }}{{\overline{R}_{2019} }} - \frac{{\overline{Y}_{1990} }}{{\overline{R}_{1990} }} = E + C = \mathop \sum \limits_{k = 1}^{K} W_{{\Delta X_{k} }} E + \mathop \sum \limits_{k = 1}^{K} W_{{\Delta \beta_{k} }} C = \mathop \sum \limits_{k = 1}^{K} E_{k} + \mathop \sum \limits_{k = 1}^{K} C_{k}$$

We chose 1990 as the comparison group and 2019 as the reference group. Therefore, endowments provide a counterfactual comparison of the outcome difference from the perspective of 1990 (showing the expected difference if 1990 had the same covariate distribution as 2019). The coefficients provide a counterfactual comparison of outcomes from a 2019 perspective (showing the expected difference if 2019 exhibited the same behavioral responses to X as in 1990). The “mvdcmp” command in STATA 17.0 was employed for non-linear multivariate decomposition analysis^16^. Geographical maps were created in ArcGIS version 10.3 (Esri, Redlands, CA, USA).

### Ethical approval

The study was reviewed and approved by the ethics committee of Hamadan University of Medical Sciences, Hamadan, Iran (Ethical code: IR.UMSHA.REC.1402.626).

## Results

**Figure 1:**
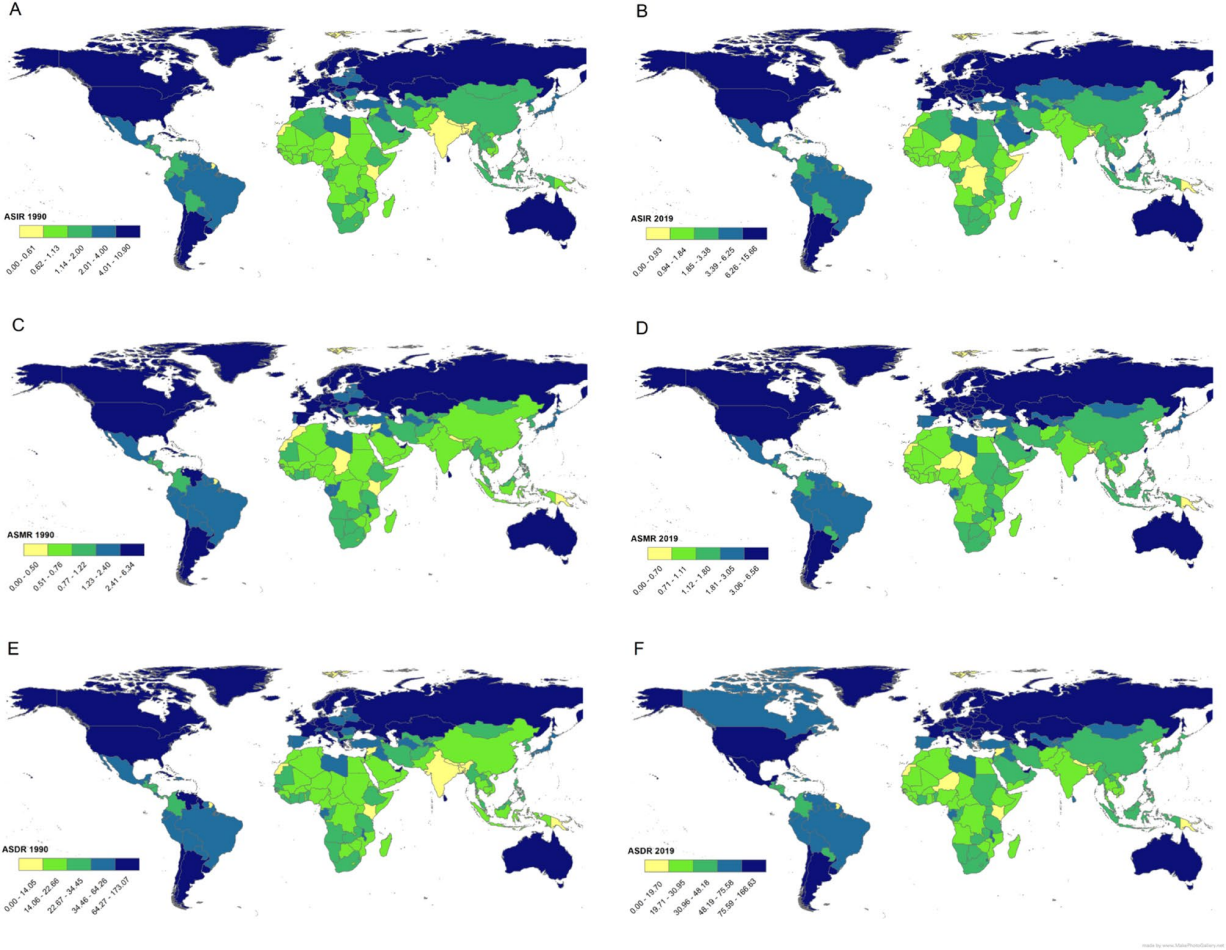
Geographical distribution of ASIR, ASMR and ASDR of kidney cancer per 100,000 people in 1990 and 2019; the Figure have been originally created by the authors in the ArcGIS version 10.3 (Esri, Redlands, CA, USA) using the available public use data (https://vizhub.healthdata.org/gbd-results/).

The geographical distribution of the ASIR, ASMR and ASDR of KC in 1990 and 2019 is shown in Fig. 1. The pattern of KC observed between 1990 and 2019 shows that KC distributed unevenly, with the burden of KC being higher in the countries in Europe, North America and Australia, and lower in the countries in Asia and Africa. The most significant changes in the KC ASIR, ASMR, and ASDR values between 1990 and 2019 were observed in Eastern European countries, while most African countries showed the smallest change in these three burden metrics. In the some countries and territory (e.g., Saint Kitts and Nevis, Bermuda, Trinidad and Tobago, Austria and Sweden), these three measures decreased between 1990 and 2019.

The mean of ASIR in 1990 was 2.88 per 100,000 people, and 4.37 per 100,000 people in 2019. In 1990, the countries with the lowest ASIR were Kenya (0.48 per 100,000 people), Nepal (0.49), and Bangladesh and Bhutan (0.60). The highest ASIR was in USA (10.90), the Czech Republic (10.18) and Iceland (10.09), respectively (Fig. 1A). In 2019, the countries with the lowest ASIR were Niger (0.74), Papua New Guinea (0.79) and Bangladesh (0.86), and the highest were the Czech Republic (12.54), Estonia (12.96) and Iceland (12.54). In 1990 and 2019, the ASIR in Estonia increased from 3.79 to 12.96 (∆change = 9.16), in Latvia from 3.48 to 12.09 (8.61) and in Belarus from 2.61 to 11.17 (8.56) (Fig. 1B).

In 1990, the mean ASMR was 1.70 per 100,000 people, while in 2019 it was 2.16 per 100,000 people. In 1990, Kenya (0.39 per 100,000 people), Papua New Guinea (0.40), and Nepal (0.42) had the lowest ASMR. Uruguay (6.34), Argentina (5.65) and Sweden (5.26) had the highest ASMR (Fig. 1C). In 2019, the ASMR was lowest in Papua New Guinea (0.49), Niger (0.55) and Bangladesh (0.59), while highest in Uruguay (6.56), Czech Republic (6.42) and Greenland (6.31). In 1990 and 2019, the $\Delta$_change_ of ASMR in Estonia (from 2.03 to 5.63), Poland (from 1.82 to 5.33) and Latvia (from 1.71 to 5.17) were 3.60, 3.51 and 3.46, respectively (Fig. 1D).

The mean ASDR per 100,000 people in 1990 and 2019 was 46.13 and 54.96. In 1990, Kenya (10.50), Nepal (11.42) and Papua New Guinea (12.20) had the lowest ASDR, while Uruguay (173.07), Argentina (160.56) and Saint Kitts and Nevis had the highest (138.81). In 2019, Papua New Guinea (14.81), Bangladesh (15.62) and Niger (16.94) had the lowest ASDR, while Uruguay (166.63), Greenland (148.46) and the Czech Republic had the highest ASDR (144.85) (Fig. 1E). In 1990 and 2019, the $\Delta$_change_ of ASDR in Belarus (from 38.21 to 120.65), Lithuania (from 54.82 to 134.17) and Latvia (from 46.94 to 125.34) were 82.44, 79.34 and 78.40, respectively (Fig. 1F). The values of ASIR, ASMR and ASDR for each country are presented in detail in the Supplementary Tables 1 and 2.

**Figure 2:**
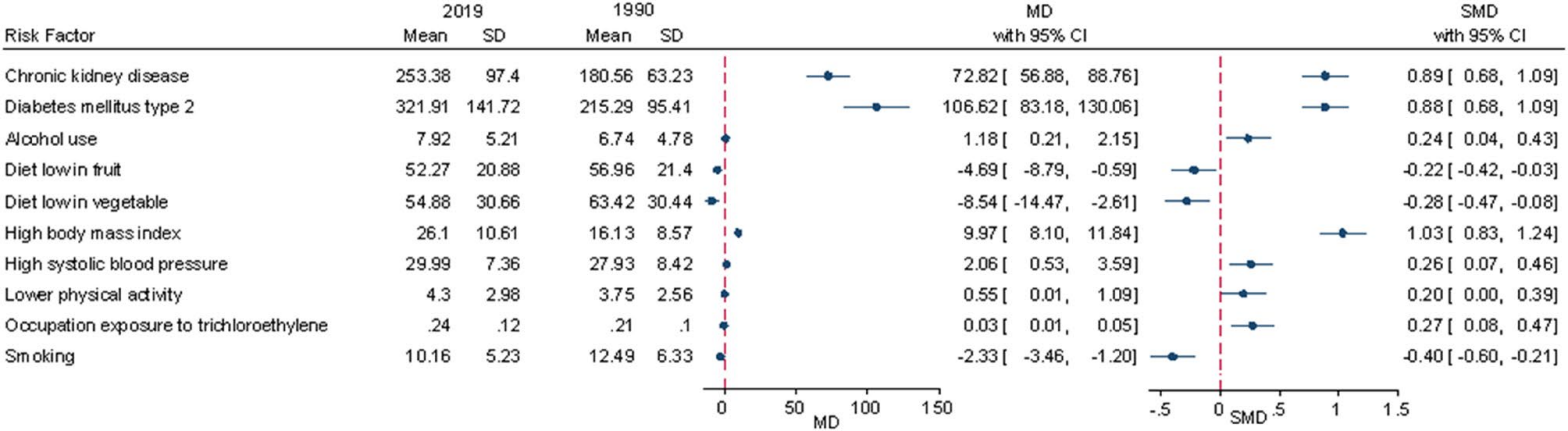
The summary statistics of the risk factors of kidney cancer in 1990 and 2019.

The distribution of risk factors from 1990 to 2019 is shown in Fig. 2. An increasing trend was observed for CKD, T2DM, high SBP, high BMI, occupational trichlorethylene exposure, low physical activity and alcohol use. The trend of fruit and vegetable consumption and smoking was decreasing. The most changes occurred for high BMI (standardized mean difference = 1.03), CKD (0.89) and T2DM (0.88). Summary statistics of KC risk factors in 1990 and 2019 are presented in detail in the Supplementary Table 3.

The adjusted effect of each risk factor on the ASIR, ASMR and ASDR of KC in the 1990 and 2019 are shown in the supplementary Table 4. High BMI and alcohol use were significantly associated with the ASIR and ASMR of KC in 1990 and 2019. For example, in 2019, for one unit increase in high BMI, the ASIR and ASMR increased by approximately 2% (incidence rate ratio = 1.017, *p*-value < 0.001) and 1% (1.015, 0.03), respectively. In the 1990 and 2019, alcohol use, diet low in vegetable, high BMI, lower physical activity, occupation exposure to trichloroethylene and smoking were significant risk factors of the ASDR of KC.

**Figure 3:**
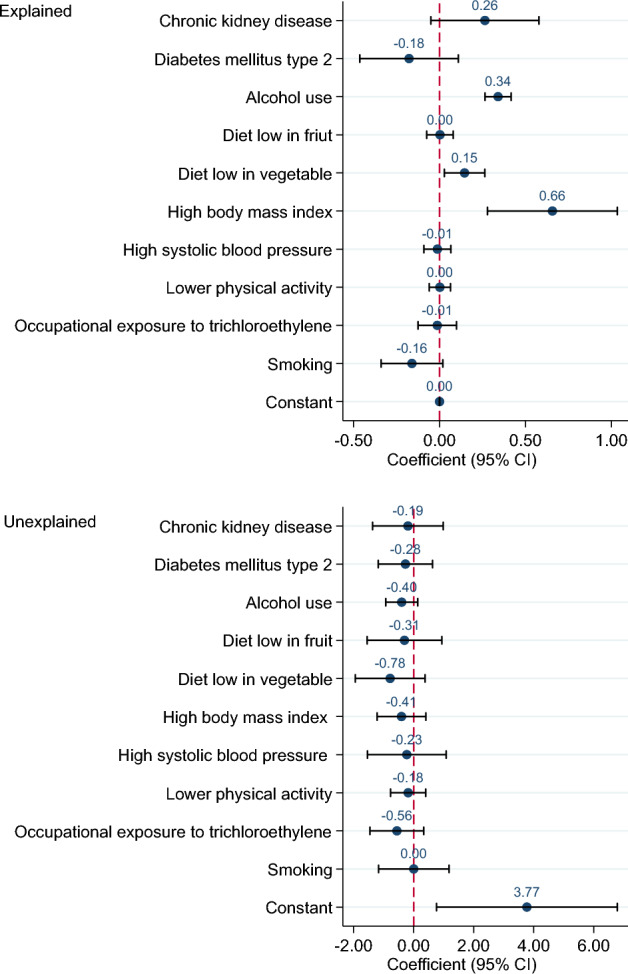
Difference decomposition of age standardized incidence rate of kidney cancer between 1990 and 2019.

**Figure 4:**
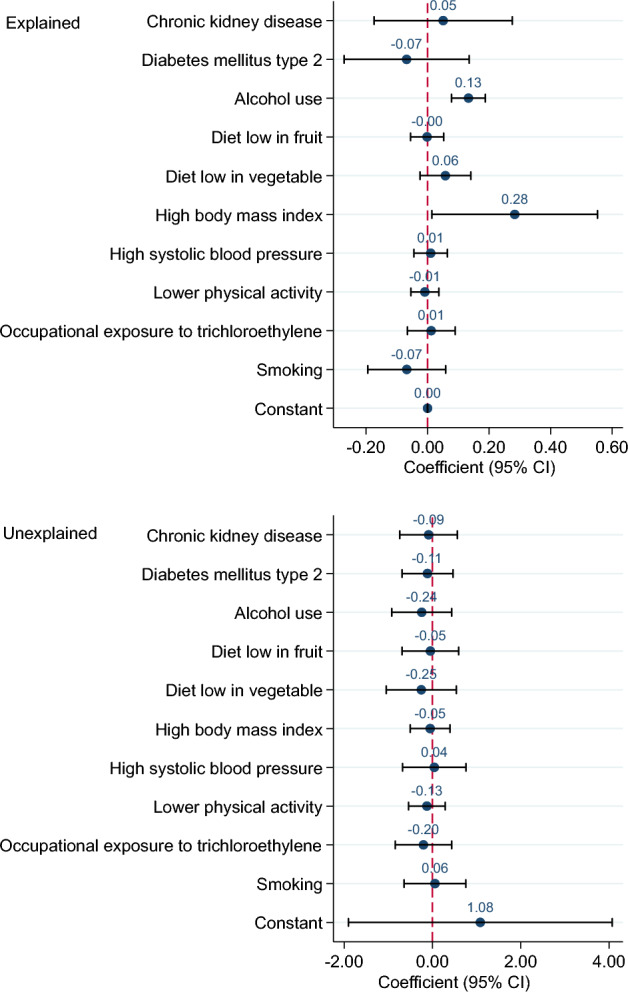
Difference decomposition of age standardized mortality rate of kidney cancer between 1990 and 2019.

**Figure 5:**
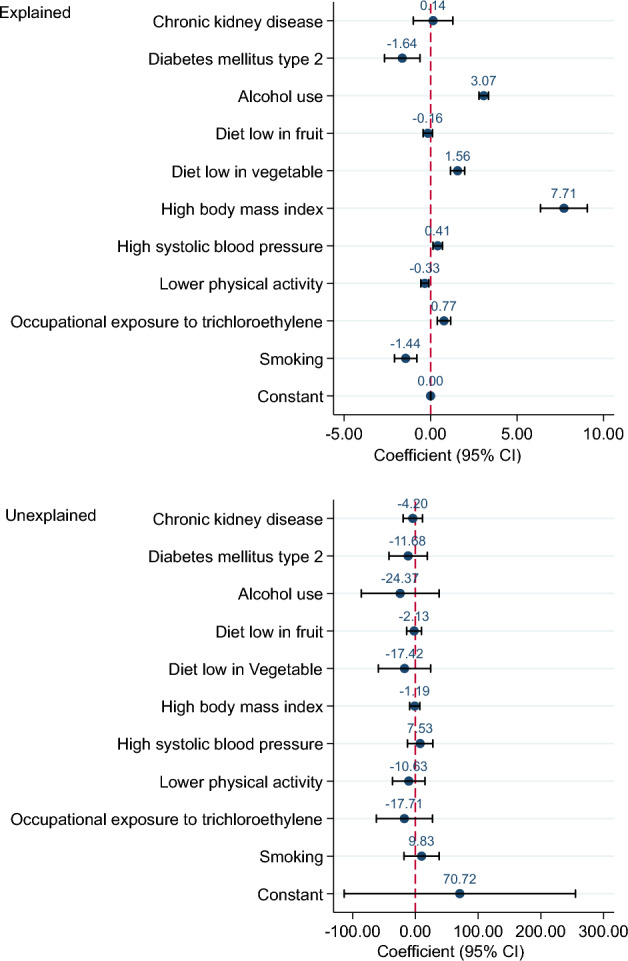
Difference decomposition of age standardized DALY rate of kidney cancer between 1990 and 2019.

Overall decomposition of difference in the ASIR, ASMR and ASDR of KC between 1990 and 2019 is shown in Table 1. Differences in characteristics significantly account for about 70, 87 and 114% of the observed time differential in the ASIR, ASMR and ASDR of KC, respectively. However, the differences in the effects were non-significant. The impact of each risk factor on the difference in ASIR of KC is shown in Fig. 3. High BMI (coefficient = 0.66, *p*-value = 0.001) and alcohol use (0.34, < 0.001) had significant impact on the changes of ASIR of KC from 1990 to 2019. In other words, high BMI and alcohol use were responsible for 44.16% and 22.90% of the difference in the ASIR. For example, if the high BMI remained constant over time, it was expected that the difference in the ASIR of KC between 1990 and 2019 would reduce by about 44%. Figure 4 shows difference decomposition of ASMR of KC between 1990 and 2019. High BMI and alcohol use account significantly for 61.67% (coefficient = 0.28, *p*-value = 0.04) and 28.95% (0.13, < 0.001) of the observed difference in the ASMR of KC. The impact of each risk factor on the difference in ASDR of KC is shown in Fig. 5. T2DM, alcohol use, diet low in vegetable, high BMI, high SBP, lower physical activity, occupation exposure to trichloroethylene and smoking had significant impact on the time differential in the ASDR of KC, however, high BMI (87.30%) and alcohol use (34.75) had greatest contribution. The results of decomposition analysis are presented in detail in the supplementary Table 5.

**Table 1:** Overall multivariate decomposition analysis.

	Coefficient	95% CI	*p*-value	Percentage
ASIR				
Endowments	1.04	0.60, 1.49	< 0.001	70.34
Coefficients	0.44	− 0.10, 0.98	0.11	29.66
Mean difference (Mean_2019_: 4.37, Mean_1990_:2.88)	1.49	1.11, 1.85	< 0.001	
ASMR				
Endowments	0.40	0.08, 0.72	0.01	87.34
Coefficients	0.06	− 0.34, 0.45	0.77	12.66
Mean difference (Mean_2019_: 2.16, Mean_1990_: 1.70)	0.46	0.19, 0.73	0.001	
ASDR				
Endowments	10.09	8.25, 11.66	< 0.001	114.23
Coefficients	− 1.26	− 3.25, 0.74	0.22	− 14.23
Mean difference (Mean_2019_: 54.96, Mean_1990_: 46.13)	8.83	7.45, 10.21	< 0.001

The mean of SDI in 1990 and 2019 was 0.48 $\pm$ 0.19 and 0.64 $\pm$ 0.17, respectively. After entering the SDI in the model, the results showed that the SDI has the most significant role in the inequality of KC burden over time. For example, about 77, 82, and 66% of the difference in the ASIR, ASMR, and ASDR between 1990 and 2019 are due to SDI, respectively. Among all other risk factors, the alcohol use only had a significant effect on the difference of ASIR (9.84%) and ASMR (16.03%), however, except for CKD and diet low in fruit, all other risk factors had an effect on the difference in the ASDR. The results are provided in detail in the supplementary Table 6.

## Discussion

We aimed to decompose the mean difference of the KC burden measures between 1990 and 2019. The pattern of KC observed between 1990 and 2019 shows that KC was unevenly distributed, with higher burdens in European, North American and Australian countries and lower in Asian and African countries. The most significant changes in the KC burden measures between 1990 and 2019 were observed in Eastern European countries. More than 70% of the changes in mean difference of KC burden measures between 1990 and 2019 are explained by changes in the risk factors. SDI, high BMI and alcohol consumption had the greatest contribution.

The present study indicates that the KC burden measures have shown an increasing trend over the past 30 years. The highest and lowest rates of ASIR, ASMR and ASDR have occurred in developed and developing or less developed countries, respectively. This increasing trend in the KC burden measures may affected by age, period and cohort (APC) effects, leading to the change in lifestyle and exposure to the risk factors of KC, as well as changes in the diagnostic processes of kidney tumors over time^22^.

The prediction models suggest that in the next decade, the trend of KC will be flat or decreasing in the developed countries, and an increasing trend is projected in the developing and less developed countries^3^. One reason for the high incidence of KC in the developed countries in recent decades may be overdiagnosis. This means that with the increasing use of advanced diagnostic methods, even small kidney masses are detected. It has been suggested that up to 50% of the increase in KC incidence in the developed countries is due to overdiagnosis^23,24^. It has been mentioned that the decrease in the incidence of KC in the developed countries in the next decade can be explained by the change in risk factors and also by the less use of unnecessary diagnostic methods^3^. The increase in the KC incidence in the less developed countries is attributed to increased ascertainment rate of KC^24^, demographic changes such as aging^1,25^, adopting Western lifestyle^6,26^, and rising prevalence of well-known risk factors for KC, such as CKD^27^, obesity^28,29^, and smoking^4^.

It's possible that the patterns of KC burden measures and its risk factors within countries may be different from those at a higher regional level. For example, in a study in China with an SDI of 0.69, it has been shown that the trend of KC incidence and mortality is still increasing in the next decade. Smoking, high BMI and aging may be possible causes of increased KC in China^30^. Since the KC burden are expected to increase in the coming years in less developed and developing countries, the health systems in these countries should take steps to provide the necessary resources to manage the treatment of KC.

In this study, SDI was assigned the most significant role in the mean difference in KC burden measures. The previous study^12^ showed a strong positive correlation between KC incidence and mortality and SDI in all years from 1990 to 2019. For example, 75% of the variation in KC incidence was due to changes in SDI (correlation coefficient 0.8675). The association between SDI and KC measures was stronger than the association of SDI with other urogenital cancers^12^. The positive correlation between the incidence of KC and SDI can be explained by the impact of socioeconomic on access to diagnostic interventions^24^, changes in risk factors^4,27^, as well as higher percentage of childhood KC in developed countries^31^. The correlation between KC mortality and SDI may be justified with higher rates of KC in the racial groups, such as blacks in the developed countries^32^, which may have impact on accessibility to treatment interventions. The low incidence of KC in less developed countries may be due to lack of health awareness, lack of routine medical and diagnostic care, and lack of specialists such as nephrologists and oncologists^33^.

In this study, we found that among the risk factors of KC, high BMI is one of the risk factors that have changed the most between 1990 and 2019, and about 44, 62 and 87% of the difference in the ASIR, ASMR and ASDR of KC between 1990 and 2019 is explained by high BMI. In the countries with higher SDI, high BMI is a main contributor for CKD-T2DM DALY burden^34^. An interaction between BMI and hypertension for increased risk of KC is well established^35^. Thus, in the developed nation and regions, interaction between high BMI, metabolic syndrome and CKD can be considered as one of main reason for increasing rate of KC incidence. A previous study found that in 2017, 18.5% of KC is due to high BMI, which could rise to 29% in high-income North America^2^. Since it has been established that the highest burden of obesity and weight gain occurs in the Eastern Mediterranean countries with medium SDI^36^, the KC rate in these regions can be explained by the high BMI in the coming years.

Our study found that alcohol use was another important risk factor explaining the variation in KC burden measures between 1990 and 2019 and also the mean alcohol consumption also had an increasing trend during the study period (standardized mean difference = 0.24). Most of the population that consumes alcohol harmfully is in the developed countries and in the age group of 15–39. Thus, in the developed countries, one solution to reduce KC in the future may be the implementation of programs to prevent alcohol consumption among younger adults. This task is even more important when we know that alcohol consumption is increasing in the next years^37^.

The current study has several limitations. It is important to emphasize that global interpretation may be prone to ecological bias, and the trend of KC and its risk factors should be analyzed within countries and at subnational levels. APC effects may have influenced the results, and our analysis was not adjusted for APC effects. Finally, the mvdcmp Stata package provides only a standard two-way decomposition, reporting differences due to characteristics and coefficient, however, interaction caused by the simultaneous difference of groups or times in the endowment and coefficient may also affect the temporal difference.

In summary, it can be concluded that more than 70% of the changes in the KC burden measures between 1990 and 2019 can be explained by its risk factors, especially SDI, high BMI and alcohol use. Based on demographic changes such as aging and the continued increase in risk factors for KC, the provision of resources for KC risk lifestyle modification and patient access to diagnostic and therapeutic modalities is essential for the treatment and management of KC.

### Supplementary Information

Supplementary Tables.

## Data Availability

The datasets analyzed during the current study are publicly available at https://vizhub.healthdata.org/gbd-results/.

## Abbreviations

KC: Kidney cancer
GBD: Global burden of disease
DALY: Disability adjusted life-year
ASIR: Age-standardized incidence rate
ASMR: Age-standardized mortality rate
ASDR: Age-standardized DALY rate
SDI: Socio-demographic index
CKD: Chronic kidney diseases
IHME: Institute for Health Metrics and Evaluation
SEV: Summary exposure value
T2DM: Type 2 diabetes mellitus
GHDx: Global health data exchange
BMI: Body mass index
SBP: Systolic blood pressure
