# Supplementary material for: Decomposing difference in the kidney cancer burden measures between 1990 and 2019 based on the global burden of disease study
Source: Sci Rep. 2024 May 6;14:10390. doi: 10.1038/s41598-024-61300-2 (PMC11074136; doi:10.1038/s41598-024-61300-2)
Supplement: Supplementary file 1 — Supplementary Tables. [file 41598_2024_61300_MOESM1_ESM.docx]

**Supplementary files**

| **Table 1.** The values of age-standardized incidence rate, age-standardized mortality rate and age-standardized DALY rate of kidney cancer and its risk factors for each country in 1990 | | | | | | | | | | | | | | |
| --- | --- | --- | --- | --- | --- | --- | --- | --- | --- | --- | --- | --- | --- | --- |
| Country | ASIR | ASMR | ASDR | Chronic kidney disease | Diabetes mellitus type 2 | Alcohol use | Diet low in fruit | Diet low in vegetable | High body mass index | High systolic blood pressure | Lower physical activity | Occupation exposure to trichloroethylene | Smoking | SDI |
| Afghanistan | 1.13 | 0.83 | 24.97 | 242.22 | 226.88 | 0.00 | 72.95 | 98.27 | 8.05 | 21.79 | 6.49 | 0.18 | 3.01 | 0.19 |
| Albania | 2.18 | 1.52 | 37.96 | 129.86 | 106.83 | 3.46 | 54.16 | 37.55 | 16.95 | 41.49 | 1.96 | 0.03 | 15.71 | 0.54 |
| Algeria | 1.26 | 0.72 | 19.05 | 270.77 | 211.89 | 0.74 | 60.24 | 57.75 | 17.38 | 28.59 | 7.27 | 0.15 | 11.49 | 0.44 |
| American Samoa | 1.53 | 0.85 | 22.03 | 214.94 | 541.12 | 2.20 | 47.34 | 78.48 | 44.53 | 27.12 | 6.11 | 0.37 | 14.02 | 0.61 |
| Andorra | 6.38 | 3.44 | 79.93 | 221.99 | 126.55 | 17.18 | 37.54 | 26.47 | 25.05 | 35.03 | 5.34 | 0.08 | 21.20 | 0.83 |
| Angola | 0.92 | 0.78 | 23.78 | 87.50 | 207.59 | 3.90 | 76.23 | 98.74 | 4.14 | 32.43 | 3.21 | 0.43 | 5.83 | 0.24 |
| Antigua and Barbuda | 3.68 | 2.26 | 63.82 | 209.52 | 313.99 | 4.10 | 35.68 | 67.56 | 14.18 | 23.01 | 4.63 | 0.39 | 4.99 | 0.58 |
| Argentina | 8.56 | 5.65 | 160.57 | 213.34 | 172.35 | 15.19 | 45.52 | 59.85 | 12.73 | 13.49 | 0.80 | 0.37 | 18.71 | 0.58 |
| Armenia | 1.14 | 0.73 | 20.17 | 103.27 | 156.94 | 4.98 | 50.08 | 7.16 | 16.10 | 36.85 | 1.64 | 0.19 | 18.36 | 0.54 |
| Australia | 7.23 | 3.70 | 87.72 | 253.36 | 127.71 | 15.26 | 59.38 | 50.23 | 22.88 | 28.04 | 4.67 | 0.08 | 19.30 | 0.74 |
| Austria | 8.67 | 4.84 | 113.52 | 217.34 | 122.47 | 16.58 | 40.95 | 56.09 | 16.34 | 31.67 | 4.49 | 0.07 | 17.78 | 0.75 |
| Azerbaijan | 4.60 | 2.96 | 93.79 | 118.42 | 130.57 | 7.23 | 49.75 | 22.92 | 19.13 | 32.27 | 1.69 | 0.26 | 13.31 | 0.58 |
| Bahamas | 4.34 | 2.66 | 80.80 | 192.18 | 287.53 | 10.19 | 50.52 | 27.06 | 26.17 | 24.05 | 5.36 | 0.29 | 5.78 | 0.69 |
| Bahrain | 3.44 | 2.34 | 55.23 | 305.90 | 442.46 | 3.95 | 25.73 | 18.26 | 30.62 | 29.38 | 8.86 | 0.27 | 12.07 | 0.55 |
| Bangladesh | 0.60 | 0.50 | 13.85 | 93.23 | 166.27 | 0.11 | 95.62 | 99.96 | 2.25 | 17.78 | 2.15 | 0.23 | 12.96 | 0.27 |
| Barbados | 5.70 | 3.45 | 98.92 | 186.01 | 319.00 | 8.49 | 76.14 | 71.44 | 27.49 | 20.76 | 6.10 | 0.34 | 6.32 | 0.65 |
| Belarus | 2.61 | 1.31 | 38.21 | 108.08 | 110.86 | 12.09 | 68.72 | 38.93 | 17.37 | 39.88 | 1.67 | 0.07 | 17.34 | 0.59 |
| Belgium | 6.52 | 3.70 | 86.02 | 232.59 | 146.25 | 16.25 | 57.35 | 38.08 | 16.61 | 22.00 | 5.47 | 0.06 | 21.54 | 0.75 |
| Belize | 2.91 | 1.87 | 59.06 | 186.62 | 210.92 | 6.49 | 11.76 | 82.49 | 16.87 | 20.09 | 4.66 | 0.27 | 7.74 | 0.43 |
| Benin | 0.93 | 0.72 | 24.07 | 137.16 | 155.88 | 2.88 | 81.43 | 71.57 | 8.59 | 24.52 | 2.31 | 0.31 | 4.69 | 0.21 |
| Bermuda | 7.87 | 4.61 | 123.68 | 181.62 | 198.66 | 13.54 | 35.74 | 27.28 | 33.23 | 21.61 | 6.11 | 0.39 | 7.55 | 0.69 |
| Bhutan | 0.60 | 0.51 | 14.05 | 127.77 | 152.47 | 3.77 | 81.23 | 87.26 | 5.43 | 20.57 | 3.33 | 0.22 | 5.37 | 0.23 |
| Bolivia (Plurinational State of) | 1.70 | 1.42 | 39.43 | 167.13 | 191.66 | 6.07 | 46.48 | 79.66 | 16.66 | 11.78 | 2.03 | 0.29 | 6.76 | 0.41 |
| Bosnia and Herzegovina | 3.08 | 2.01 | 53.09 | 129.00 | 189.29 | 7.50 | 70.43 | 37.05 | 15.19 | 33.57 | 1.98 | 0.04 | 17.63 | 0.53 |
| Botswana | 1.08 | 0.86 | 22.66 | 153.49 | 218.41 | 5.94 | 91.11 | 90.98 | 10.42 | 32.33 | 2.97 | 0.20 | 9.32 | 0.43 |
| Brazil | 2.25 | 1.50 | 44.60 | 191.42 | 243.71 | 6.58 | 45.17 | 79.44 | 14.85 | 27.12 | 12.02 | 0.28 | 19.05 | 0.49 |
| Brunei Darussalam | 4.10 | 2.98 | 70.25 | 306.92 | 577.25 | 3.76 | 62.85 | 65.84 | 6.59 | 31.35 | 3.86 | 0.07 | 17.99 | 0.68 |
| Bulgaria | 2.00 | 1.14 | 33.30 | 143.17 | 181.77 | 12.83 | 44.41 | 16.16 | 26.11 | 35.03 | 2.16 | 0.06 | 27.43 | 0.63 |
| Burkina Faso | 0.75 | 0.58 | 19.41 | 122.21 | 165.92 | 6.95 | 99.44 | 92.69 | 5.21 | 23.35 | 1.36 | 0.20 | 3.88 | 0.13 |
| Burundi | 0.98 | 0.85 | 24.42 | 92.78 | 159.25 | 9.43 | 36.80 | 95.39 | 5.62 | 21.51 | 0.97 | 0.31 | 5.97 | 0.20 |
| Cabo Verde | 0.68 | 0.51 | 14.32 | 115.36 | 152.25 | 4.87 | 79.25 | 82.11 | 8.99 | 35.45 | 2.08 | 0.17 | 4.70 | 0.29 |
| Cambodia | 1.11 | 0.75 | 22.08 | 116.69 | 151.34 | 1.64 | 86.81 | 91.11 | 2.84 | 19.41 | 1.57 | 0.27 | 13.50 | 0.27 |
| Cameroon | 0.70 | 0.57 | 16.58 | 180.09 | 150.22 | 5.92 | 35.30 | 73.58 | 17.37 | 15.70 | 2.64 | 0.25 | 5.03 | 0.31 |
| Canada | 7.39 | 2.61 | 65.04 | 239.66 | 112.42 | 12.88 | 47.20 | 33.40 | 22.60 | 22.71 | 3.42 | 0.09 | 24.22 | 0.79 |
| Central African Republic | 0.87 | 0.76 | 22.15 | 89.65 | 206.49 | 5.69 | 67.60 | 99.96 | 6.28 | 31.57 | 3.43 | 0.23 | 4.31 | 0.19 |
| Chad | 0.61 | 0.48 | 16.28 | 127.15 | 151.85 | 2.36 | 91.83 | 99.96 | 3.65 | 23.23 | 1.84 | 0.18 | 4.66 | 0.11 |
| Chile | 6.13 | 4.12 | 107.62 | 214.27 | 191.14 | 12.35 | 61.14 | 44.97 | 18.58 | 27.89 | 2.81 | 0.29 | 18.26 | 0.59 |
| China | 1.16 | 0.70 | 21.59 | 146.38 | 174.27 | 5.41 | 74.00 | 39.99 | 4.70 | 19.15 | 2.14 | 0.30 | 15.23 | 0.43 |
| Colombia | 1.77 | 1.20 | 34.42 | 221.92 | 296.36 | 7.85 | 45.84 | 65.49 | 13.52 | 18.13 | 2.20 | 0.32 | 10.47 | 0.48 |
| Comoros | 1.06 | 0.92 | 25.09 | 100.50 | 146.44 | 0.51 | 36.40 | 99.96 | 12.42 | 27.33 | 0.79 | 0.24 | 5.65 | 0.27 |
| Congo | 1.44 | 1.23 | 34.45 | 102.32 | 220.82 | 6.38 | 65.74 | 98.73 | 10.31 | 34.89 | 3.75 | 0.23 | 4.01 | 0.36 |
| Cook Islands | 1.46 | 0.63 | 17.61 | 181.84 | 390.07 | 2.49 | 51.33 | 82.11 | 33.87 | 17.22 | 4.44 | 0.33 | 12.20 | 0.63 |
| Costa Rica | 2.25 | 1.40 | 36.17 | 436.12 | 270.02 | 8.29 | 36.27 | 90.21 | 22.91 | 36.13 | 1.30 | 0.30 | 11.73 | 0.53 |
| Croatia | 2.96 | 1.54 | 39.62 | 164.96 | 206.90 | 12.70 | 51.18 | 35.74 | 20.28 | 42.73 | 2.01 | 0.05 | 26.51 | 0.68 |
| Cuba | 4.61 | 2.48 | 75.39 | 142.07 | 276.82 | 7.04 | 45.04 | 60.19 | 22.43 | 21.31 | 5.08 | 0.27 | 17.32 | 0.58 |
| Cyprus | 1.75 | 1.12 | 26.21 | 270.13 | 326.04 | 12.03 | 30.81 | 43.92 | 13.10 | 31.16 | 3.41 | 0.07 | 19.82 | 0.66 |
| Czechia | 10.18 | 5.10 | 131.47 | 144.18 | 254.07 | 16.50 | 55.44 | 49.17 | 20.75 | 35.98 | 2.44 | 0.09 | 19.95 | 0.69 |
| Côte d'Ivoire | 1.07 | 0.85 | 26.22 | 154.50 | 161.83 | 5.65 | 47.29 | 78.43 | 9.40 | 34.68 | 2.56 | 0.24 | 5.69 | 0.26 |
| Democratic People's Republic of Korea | 2.03 | 0.82 | 26.78 | 152.02 | 134.07 | 5.25 | 53.65 | 19.49 | 3.84 | 36.20 | 1.85 | 0.26 | 10.22 | 0.43 |
| Democratic Republic of the Congo | 0.86 | 0.73 | 21.04 | 93.09 | 194.07 | 4.37 | 54.00 | 99.96 | 8.59 | 34.55 | 3.48 | 0.28 | 4.15 | 0.26 |
| Denmark | 4.37 | 2.79 | 65.58 | 197.94 | 102.78 | 15.67 | 59.62 | 52.32 | 15.72 | 34.50 | 4.21 | 0.09 | 25.57 | 0.81 |
| Djibouti | 1.06 | 0.89 | 24.66 | 98.71 | 150.72 | 2.39 | 96.25 | 82.62 | 5.26 | 22.40 | 0.98 | 0.19 | 7.72 | 0.28 |
| Dominica | 4.29 | 2.76 | 79.23 | 230.14 | 327.73 | 8.77 | 4.82 | 52.87 | 22.32 | 24.07 | 4.43 | 0.25 | 6.94 | 0.58 |
| Dominican Republic | 2.19 | 1.45 | 49.05 | 129.19 | 132.22 | 7.46 | 23.53 | 78.23 | 12.30 | 21.72 | 7.13 | 0.33 | 8.67 | 0.43 |
| Ecuador | 1.86 | 1.41 | 39.53 | 171.94 | 183.29 | 4.24 | 14.81 | 92.32 | 27.18 | 11.03 | 2.57 | 0.36 | 7.51 | 0.50 |
| Egypt | 1.06 | 0.61 | 19.08 | 275.60 | 140.81 | 0.48 | 42.63 | 5.17 | 23.83 | 20.29 | 7.67 | 0.16 | 11.18 | 0.40 |
| El Salvador | 1.37 | 0.97 | 28.79 | 228.21 | 205.96 | 4.47 | 59.62 | 87.81 | 15.25 | 24.24 | 1.21 | 0.28 | 5.91 | 0.39 |
| Equatorial Guinea | 0.77 | 0.68 | 19.70 | 87.55 | 205.25 | 4.18 | 68.01 | 99.32 | 5.36 | 31.23 | 3.29 | 0.16 | 4.24 | 0.21 |
| Eritrea | 0.78 | 0.69 | 19.85 | 87.67 | 153.93 | 2.33 | 75.45 | 97.70 | 3.72 | 17.88 | 1.04 | 0.24 | 3.98 | 0.20 |
| Estonia | 3.79 | 2.03 | 53.22 | 130.31 | 122.39 | 12.32 | 67.85 | 56.73 | 20.63 | 38.65 | 1.50 | 0.09 | 17.65 | 0.67 |
| Eswatini | 1.35 | 1.10 | 30.01 | 181.59 | 256.57 | 5.38 | 74.81 | 91.50 | 23.67 | 27.80 | 3.30 | 0.21 | 4.15 | 0.39 |
| Ethiopia | 1.22 | 1.10 | 29.39 | 93.76 | 170.71 | 3.24 | 99.23 | 98.95 | 4.06 | 9.32 | 1.10 | 0.27 | 2.44 | 0.14 |
| Fiji | 1.14 | 0.66 | 18.15 | 199.10 | 529.21 | 3.26 | 87.74 | 88.36 | 23.51 | 23.77 | 4.12 | 0.28 | 13.85 | 0.53 |
| Finland | 8.49 | 4.13 | 99.08 | 161.76 | 193.63 | 13.58 | 60.74 | 62.73 | 18.71 | 50.68 | 4.15 | 0.08 | 17.75 | 0.76 |
| France | 6.90 | 3.89 | 94.24 | 199.04 | 93.26 | 17.42 | 54.10 | 39.83 | 15.75 | 32.10 | 7.08 | 0.07 | 18.54 | 0.74 |
| Gabon | 1.62 | 1.31 | 36.98 | 118.24 | 237.36 | 10.83 | 17.30 | 88.01 | 11.79 | 27.27 | 3.02 | 0.16 | 3.96 | 0.39 |
| Gambia | 0.70 | 0.55 | 16.60 | 132.82 | 138.67 | 2.52 | 99.98 | 95.28 | 7.88 | 28.39 | 2.20 | 0.22 | 8.42 | 0.22 |
| Georgia | 3.41 | 1.94 | 58.48 | 113.71 | 139.91 | 5.62 | 50.88 | 24.45 | 25.11 | 49.95 | 1.71 | 0.26 | 14.36 | 0.65 |
| Germany | 8.63 | 3.81 | 97.05 | 237.09 | 242.26 | 18.37 | 42.82 | 49.67 | 19.22 | 40.13 | 3.10 | 0.08 | 21.21 | 0.82 |
| Ghana | 1.21 | 0.86 | 30.44 | 120.98 | 164.97 | 4.91 | 48.99 | 79.49 | 6.96 | 29.45 | 2.16 | 0.53 | 3.18 | 0.36 |
| Greece | 5.27 | 2.89 | 67.21 | 272.66 | 144.49 | 14.03 | 14.43 | 2.57 | 21.24 | 29.72 | 2.51 | 0.06 | 24.85 | 0.68 |
| Greenland | 5.80 | 3.93 | 100.47 | 197.20 | 100.61 | 10.12 | 46.90 | 31.47 | 22.93 | 21.55 | 2.88 | 0.08 | 26.25 | 0.66 |
| Grenada | 2.92 | 1.93 | 57.62 | 206.41 | 327.37 | 8.96 | 32.17 | 92.35 | 12.77 | 21.25 | 4.27 | 0.27 | 5.70 | 0.46 |
| Guam | 3.94 | 1.88 | 47.71 | 180.05 | 255.86 | 2.74 | 37.95 | 69.56 | 35.56 | 19.94 | 3.63 | 0.32 | 11.46 | 0.69 |
| Guatemala | 1.46 | 1.14 | 33.19 | 237.26 | 228.59 | 4.43 | 61.45 | 77.91 | 9.77 | 15.05 | 0.65 | 0.33 | 6.02 | 0.32 |
| Guinea | 0.79 | 0.62 | 21.33 | 134.50 | 136.08 | 1.18 | 36.78 | 72.66 | 7.33 | 23.54 | 1.89 | 0.15 | 5.67 | 0.18 |
| Guinea-Bissau | 1.06 | 0.84 | 28.45 | 145.13 | 167.64 | 4.34 | 67.72 | 99.96 | 6.08 | 27.55 | 2.26 | 0.19 | 2.94 | 0.20 |
| Guyana | 3.39 | 2.39 | 73.20 | 176.43 | 381.62 | 9.20 | 61.91 | 91.49 | 13.95 | 21.00 | 4.63 | 0.28 | 7.35 | 0.45 |
| Haiti | 1.94 | 1.53 | 50.98 | 154.51 | 303.33 | 6.47 | 42.99 | 93.48 | 6.61 | 28.47 | 4.05 | 0.20 | 4.80 | 0.31 |
| Honduras | 1.18 | 0.91 | 25.80 | 227.04 | 274.42 | 3.82 | 49.60 | 86.73 | 9.74 | 24.63 | 1.12 | 0.28 | 9.33 | 0.33 |
| Hungary | 8.11 | 4.81 | 123.88 | 123.97 | 198.56 | 14.33 | 50.32 | 40.23 | 25.93 | 45.18 | 1.83 | 0.07 | 22.73 | 0.66 |
| Iceland | 10.09 | 5.20 | 120.65 | 184.21 | 118.29 | 8.49 | 65.07 | 74.55 | 23.89 | 19.26 | 5.57 | 0.10 | 21.60 | 0.76 |
| India | 0.61 | 0.51 | 13.53 | 155.42 | 193.09 | 2.29 | 97.71 | 72.06 | 4.20 | 24.05 | 2.88 | 0.21 | 8.36 | 0.33 |
| Indonesia | 1.15 | 0.69 | 21.46 | 125.66 | 156.92 | 0.70 | 80.74 | 87.61 | 4.66 | 36.40 | 2.75 | 0.31 | 11.17 | 0.45 |
| Iran (Islamic Republic of) | 1.80 | 0.95 | 26.83 | 312.23 | 170.51 | 0.24 | 23.82 | 36.17 | 15.20 | 21.51 | 6.56 | 0.18 | 10.21 | 0.40 |
| Iraq | 2.05 | 1.24 | 35.18 | 302.88 | 286.87 | 1.79 | 41.13 | 15.87 | 26.59 | 33.89 | 8.22 | 0.18 | 16.02 | 0.39 |
| Ireland | 5.47 | 3.05 | 74.89 | 264.06 | 83.59 | 14.46 | 68.12 | 53.10 | 20.86 | 36.43 | 8.32 | 0.06 | 21.77 | 0.73 |
| Israel | 4.56 | 2.96 | 66.58 | 272.65 | 169.26 | 3.74 | 16.26 | 4.61 | 20.10 | 30.54 | 5.49 | 0.06 | 20.28 | 0.72 |
| Italy | 8.97 | 3.46 | 88.77 | 210.71 | 212.97 | 15.89 | 27.81 | 10.79 | 17.64 | 37.82 | 6.93 | 0.07 | 20.09 | 0.71 |
| Jamaica | 2.78 | 1.69 | 50.50 | 197.16 | 282.00 | 5.30 | 38.04 | 65.49 | 19.50 | 15.59 | 5.87 | 0.33 | 9.47 | 0.54 |
| Japan | 3.26 | 1.79 | 42.76 | 273.87 | 156.25 | 12.91 | 63.20 | 24.89 | 9.08 | 36.98 | 4.32 | 0.09 | 20.16 | 0.79 |
| Jordan | 1.36 | 0.80 | 20.82 | 296.93 | 300.40 | 0.80 | 46.29 | 37.29 | 25.34 | 25.09 | 5.02 | 0.17 | 19.81 | 0.52 |
| Kazakhstan | 4.56 | 2.91 | 82.92 | 112.79 | 161.66 | 9.82 | 81.28 | 36.33 | 21.74 | 47.76 | 2.40 | 0.28 | 13.72 | 0.60 |
| Kenya | 0.48 | 0.39 | 10.50 | 92.61 | 130.05 | 6.17 | 69.46 | 78.78 | 10.77 | 31.18 | 0.93 | 0.36 | 5.28 | 0.33 |
| Kiribati | 3.22 | 2.19 | 66.12 | 155.16 | 389.63 | 2.39 | 61.14 | 83.76 | 20.03 | 15.28 | 4.31 | 0.10 | 17.61 | 0.43 |
| Kuwait | 3.01 | 1.22 | 36.24 | 299.90 | 346.89 | 0.00 | 39.03 | 11.55 | 37.23 | 25.11 | 11.10 | 0.26 | 16.73 | 0.66 |
| Kyrgyzstan | 1.60 | 1.04 | 31.60 | 114.54 | 102.97 | 5.35 | 78.12 | 22.03 | 17.60 | 23.36 | 1.66 | 0.26 | 15.20 | 0.53 |
| Lao People's Democratic Republic | 1.20 | 0.83 | 25.82 | 165.96 | 215.78 | 6.18 | 78.85 | 97.50 | 3.48 | 27.25 | 1.46 | 0.27 | 14.15 | 0.27 |
| Latvia | 3.48 | 1.71 | 46.94 | 111.24 | 113.82 | 11.60 | 68.34 | 32.78 | 21.72 | 41.02 | 2.49 | 0.06 | 18.40 | 0.68 |
| Lebanon | 2.09 | 1.34 | 32.91 | 271.00 | 231.30 | 2.53 | 8.12 | 2.61 | 21.51 | 25.86 | 7.79 | 0.20 | 15.48 | 0.46 |
| Lesotho | 0.74 | 0.61 | 16.59 | 149.58 | 200.15 | 5.46 | 90.95 | 91.18 | 11.15 | 24.15 | 2.15 | 0.23 | 8.22 | 0.32 |
| Liberia | 1.10 | 0.82 | 30.78 | 130.67 | 188.88 | 6.34 | 64.02 | 91.02 | 12.90 | 30.48 | 2.78 | 0.25 | 4.40 | 0.22 |
| Libya | 2.41 | 1.57 | 39.69 | 273.41 | 244.75 | 0.13 | 33.60 | 20.22 | 26.61 | 32.36 | 8.47 | 0.19 | 14.44 | 0.41 |
| Lithuania | 3.96 | 2.00 | 54.82 | 120.44 | 108.19 | 11.62 | 62.95 | 45.16 | 19.02 | 43.07 | 2.65 | 0.06 | 15.22 | 0.67 |
| Luxembourg | 3.92 | 2.22 | 53.10 | 234.10 | 102.57 | 17.82 | 49.50 | 48.31 | 18.22 | 36.13 | 4.31 | 0.06 | 20.36 | 0.82 |
| Madagascar | 0.86 | 0.72 | 21.05 | 84.90 | 138.44 | 3.46 | 65.35 | 94.85 | 6.40 | 22.44 | 0.99 | 0.26 | 6.69 | 0.27 |
| Malawi | 2.17 | 1.79 | 52.19 | 98.19 | 175.10 | 2.87 | 68.92 | 91.83 | 9.56 | 36.93 | 1.14 | 0.39 | 4.69 | 0.21 |
| Malaysia | 1.69 | 0.99 | 24.63 | 173.17 | 241.03 | 2.52 | 67.80 | 86.52 | 11.85 | 30.13 | 3.93 | 0.34 | 12.93 | 0.54 |
| Maldives | 1.34 | 0.82 | 22.02 | 191.84 | 200.81 | 0.72 | 68.00 | 71.66 | 4.09 | 30.53 | 3.68 | 0.29 | 15.48 | 0.30 |
| Mali | 0.77 | 0.61 | 20.34 | 139.41 | 143.38 | 1.11 | 80.26 | 92.28 | 4.66 | 19.89 | 1.71 | 0.19 | 2.90 | 0.13 |
| Malta | 5.09 | 3.07 | 69.96 | 244.97 | 233.83 | 9.79 | 49.66 | 31.80 | 14.28 | 37.90 | 10.89 | 0.07 | 20.25 | 0.67 |
| Marshall Islands | 1.13 | 0.74 | 20.05 | 169.25 | 499.79 | 3.25 | 67.73 | 92.57 | 12.43 | 14.01 | 5.10 | 0.19 | 9.95 | 0.40 |
| Mauritania | 1.08 | 0.88 | 25.90 | 152.02 | 126.02 | 0.04 | 94.78 | 99.95 | 14.02 | 28.48 | 9.08 | 0.17 | 6.40 | 0.31 |
| Mauritius | 1.79 | 0.83 | 23.10 | 255.00 | 304.98 | 5.87 | 89.89 | 72.42 | 12.06 | 23.46 | 3.16 | 0.28 | 10.63 | 0.53 |
| Mexico | 3.05 | 2.14 | 58.51 | 310.88 | 438.90 | 7.11 | 42.26 | 66.41 | 25.56 | 24.33 | 3.71 | 0.35 | 10.96 | 0.51 |
| Micronesia (Federated States of) | 1.44 | 0.92 | 26.16 | 193.29 | 304.31 | 4.53 | 68.01 | 92.76 | 22.78 | 13.58 | 5.46 | 0.26 | 15.26 | 0.45 |
| Monaco | 7.11 | 3.46 | 84.76 | 202.45 | 111.94 | 10.97 | 22.41 | 6.75 | 29.66 | 36.76 | 6.60 | 0.07 | 20.81 | 0.83 |
| Mongolia | 1.50 | 1.18 | 30.05 | 155.39 | 68.83 | 4.60 | 99.71 | 97.19 | 15.60 | 35.57 | 1.67 | 0.23 | 10.67 | 0.47 |
| Montenegro | 4.44 | 2.40 | 63.79 | 190.37 | 222.32 | 13.14 | 32.63 | 4.20 | 28.46 | 42.37 | 2.37 | 0.04 | 22.56 | 0.70 |
| Morocco | 0.80 | 0.50 | 14.18 | 212.54 | 178.73 | 1.29 | 50.29 | 49.76 | 16.15 | 39.67 | 7.12 | 0.24 | 10.45 | 0.35 |
| Mozambique | 0.63 | 0.54 | 16.04 | 87.30 | 154.44 | 1.47 | 89.09 | 99.95 | 5.60 | 38.10 | 0.98 | 0.24 | 4.66 | 0.12 |
| Myanmar | 1.18 | 0.78 | 23.71 | 156.36 | 240.94 | 0.99 | 88.74 | 86.82 | 2.89 | 25.90 | 1.82 | 0.15 | 17.11 | 0.28 |
| Namibia | 1.24 | 1.05 | 27.18 | 149.11 | 230.57 | 6.04 | 87.19 | 99.05 | 12.87 | 30.55 | 5.19 | 0.17 | 7.39 | 0.45 |
| Nauru | 2.63 | 1.37 | 40.22 | 193.17 | 318.93 | 5.56 | 49.45 | 80.58 | 27.85 | 28.56 | 4.16 | 0.30 | 14.76 | 0.50 |
| Nepal | 0.49 | 0.42 | 11.42 | 126.90 | 145.86 | 0.61 | 82.23 | 89.71 | 2.93 | 21.00 | 2.13 | 0.29 | 11.04 | 0.20 |
| Netherlands | 7.50 | 3.90 | 93.97 | 212.10 | 152.70 | 13.89 | 50.17 | 53.53 | 15.57 | 24.85 | 1.81 | 0.06 | 29.11 | 0.80 |
| New Zealand | 8.43 | 3.41 | 85.79 | 260.14 | 115.73 | 15.75 | 44.40 | 39.67 | 23.57 | 28.74 | 4.09 | 0.08 | 18.53 | 0.76 |
| Nicaragua | 1.45 | 0.98 | 30.93 | 270.34 | 280.55 | 4.17 | 73.27 | 99.34 | 12.41 | 29.10 | 1.18 | 0.24 | 7.93 | 0.34 |
| Niger | 0.68 | 0.53 | 18.89 | 123.65 | 94.61 | 0.70 | 97.63 | 72.41 | 5.27 | 25.72 | 2.26 | 0.19 | 2.01 | 0.07 |
| Nigeria | 0.77 | 0.60 | 21.45 | 136.83 | 137.46 | 4.99 | 60.70 | 70.83 | 8.04 | 21.56 | 2.79 | 0.25 | 2.81 | 0.31 |
| Niue | 2.08 | 0.98 | 28.84 | 193.14 | 424.00 | 5.57 | 57.60 | 86.49 | 27.60 | 24.24 | 4.30 | 0.28 | 10.87 | 0.57 |
| North Macedonia | 1.35 | 0.85 | 23.54 | 149.03 | 226.18 | 11.56 | 42.57 | 4.66 | 19.59 | 38.52 | 2.09 | 0.04 | 22.29 | 0.62 |
| Northern Mariana Islands | 3.80 | 1.65 | 44.86 | 245.15 | 301.20 | 3.37 | 39.13 | 70.61 | 45.10 | 20.20 | 4.89 | 0.31 | 13.39 | 0.69 |
| Norway | 7.50 | 3.80 | 89.71 | 162.67 | 184.48 | 10.15 | 52.30 | 63.38 | 16.63 | 43.20 | 5.24 | 0.08 | 19.42 | 0.81 |
| Oman | 1.22 | 0.69 | 18.31 | 226.36 | 241.99 | 0.61 | 29.61 | 32.59 | 13.63 | 17.23 | 7.04 | 0.21 | 9.29 | 0.44 |
| Pakistan | 0.78 | 0.66 | 17.58 | 145.29 | 185.04 | 0.68 | 72.56 | 93.46 | 6.42 | 26.37 | 3.75 | 0.16 | 11.72 | 0.25 |
| Palau | 1.23 | 0.54 | 15.91 | 221.26 | 362.71 | 3.60 | 53.72 | 83.94 | 31.12 | 16.92 | 4.36 | 0.31 | 10.21 | 0.62 |
| Palestine | 1.99 | 1.12 | 29.80 | 308.03 | 258.18 | 1.14 | 56.03 | 91.01 | 16.13 | 21.13 | 6.84 | 0.16 | 13.12 | 0.31 |
| Panama | 1.76 | 1.17 | 31.60 | 242.26 | 257.39 | 7.29 | 54.51 | 84.98 | 10.41 | 24.74 | 1.11 | 0.26 | 7.26 | 0.54 |
| Papua New Guinea | 0.62 | 0.40 | 12.20 | 89.85 | 316.34 | 3.31 | 68.50 | 73.15 | 9.57 | 9.92 | 3.57 | 0.19 | 11.03 | 0.29 |
| Paraguay | 2.15 | 1.50 | 42.32 | 188.01 | 177.73 | 11.52 | 28.95 | 79.09 | 16.65 | 26.21 | 1.89 | 0.36 | 14.84 | 0.47 |
| Peru | 2.82 | 2.08 | 59.22 | 143.66 | 126.63 | 7.93 | 60.93 | 78.05 | 16.83 | 10.64 | 2.19 | 0.46 | 3.22 | 0.50 |
| Philippines | 1.95 | 1.04 | 31.83 | 184.17 | 205.96 | 7.65 | 46.87 | 76.52 | 6.12 | 26.44 | 1.07 | 0.33 | 16.01 | 0.50 |
| Poland | 2.60 | 1.82 | 48.91 | 160.55 | 206.04 | 11.56 | 67.58 | 21.91 | 19.99 | 32.78 | 3.60 | 0.07 | 23.86 | 0.63 |
| Portugal | 4.03 | 2.07 | 57.11 | 202.63 | 220.47 | 14.06 | 42.88 | 14.43 | 14.86 | 35.79 | 6.29 | 0.08 | 15.21 | 0.61 |
| Puerto Rico | 3.64 | 2.14 | 56.03 | 222.61 | 349.31 | 8.58 | 49.94 | 93.96 | 32.54 | 24.88 | 7.04 | 0.05 | 8.07 | 0.67 |
| Qatar | 3.92 | 2.78 | 62.16 | 318.15 | 493.12 | 1.81 | 15.74 | 6.24 | 34.89 | 27.02 | 10.10 | 0.32 | 10.88 | 0.59 |
| Republic of Korea | 1.50 | 0.99 | 26.37 | 216.80 | 187.47 | 13.40 | 58.23 | 4.91 | 9.02 | 22.25 | 3.92 | 0.09 | 18.89 | 0.69 |
| Republic of Moldova | 3.52 | 1.78 | 59.13 | 84.47 | 152.34 | 15.42 | 54.35 | 22.88 | 16.12 | 37.97 | 1.63 | 0.04 | 14.46 | 0.59 |
| Romania | 3.21 | 1.82 | 56.73 | 124.86 | 134.38 | 11.95 | 54.13 | 9.45 | 19.39 | 45.93 | 2.56 | 0.09 | 18.70 | 0.63 |
| Russian Federation | 7.34 | 3.66 | 105.69 | 144.37 | 101.09 | 11.85 | 71.49 | 29.07 | 20.89 | 37.86 | 1.81 | 0.07 | 15.29 | 0.70 |
| Rwanda | 1.15 | 1.00 | 28.30 | 95.87 | 159.35 | 10.39 | 1.67 | 99.96 | 8.13 | 23.17 | 0.98 | 0.22 | 5.58 | 0.26 |
| Saint Kitts and Nevis | 7.87 | 4.77 | 138.81 | 270.10 | 356.69 | 6.21 | 83.65 | 79.26 | 21.52 | 29.06 | 4.98 | 0.40 | 4.22 | 0.58 |
| Saint Lucia | 3.34 | 2.21 | 64.26 | 198.97 | 456.23 | 11.20 | 25.21 | 91.13 | 16.84 | 26.73 | 4.66 | 0.33 | 7.91 | 0.48 |
| Saint Vincent and the Grenadines | 3.72 | 2.38 | 70.14 | 188.29 | 392.90 | 6.97 | 57.32 | 92.88 | 13.44 | 22.50 | 4.50 | 0.27 | 6.15 | 0.46 |
| Samoa | 1.61 | 0.91 | 24.58 | 191.80 | 331.84 | 4.25 | 64.93 | 99.96 | 31.94 | 18.56 | 5.26 | 0.21 | 15.90 | 0.53 |
| San Marino | 3.84 | 2.04 | 46.14 | 181.69 | 136.14 | 13.15 | 39.82 | 30.19 | 25.67 | 35.18 | 5.82 | 0.08 | 20.46 | 0.81 |
| Sao Tome and Principe | 1.13 | 0.74 | 32.84 | 168.84 | 162.26 | 4.87 | 48.69 | 95.02 | 10.99 | 37.55 | 2.20 | 0.16 | 2.39 | 0.30 |
| Saudi Arabia | 1.17 | 0.71 | 18.57 | 307.34 | 282.68 | 0.81 | 33.65 | 32.75 | 22.40 | 24.15 | 12.15 | 0.19 | 7.77 | 0.48 |
| Senegal | 0.98 | 0.76 | 25.40 | 136.93 | 213.72 | 1.11 | 86.30 | 82.06 | 9.78 | 35.62 | 3.56 | 0.21 | 6.44 | 0.23 |
| Serbia | 4.11 | 2.72 | 72.18 | 142.34 | 222.66 | 10.07 | 43.50 | 35.75 | 21.24 | 44.16 | 2.09 | 0.05 | 19.53 | 0.63 |
| Seychelles | 2.13 | 1.17 | 30.76 | 213.62 | 231.95 | 5.83 | 61.78 | 86.92 | 14.92 | 35.57 | 2.13 | 0.33 | 11.11 | 0.57 |
| Sierra Leone | 0.91 | 0.68 | 25.17 | 124.52 | 95.39 | 5.86 | 75.05 | 84.21 | 6.05 | 41.26 | 2.28 | 0.19 | 8.69 | 0.21 |
| Singapore | 3.17 | 1.95 | 47.56 | 251.88 | 267.89 | 3.75 | 47.60 | 35.11 | 7.68 | 21.89 | 3.78 | 0.08 | 11.64 | 0.69 |
| Slovakia | 5.35 | 3.06 | 79.26 | 155.94 | 173.56 | 14.61 | 64.43 | 42.78 | 18.68 | 41.34 | 2.37 | 0.07 | 18.56 | 0.66 |
| Slovenia | 5.12 | 2.44 | 61.39 | 139.78 | 197.10 | 13.67 | 40.88 | 49.17 | 21.83 | 43.72 | 2.19 | 0.07 | 20.13 | 0.73 |
| Solomon Islands | 1.08 | 0.68 | 19.70 | 160.39 | 283.04 | 2.12 | 72.46 | 99.96 | 11.52 | 11.82 | 3.43 | 0.15 | 14.80 | 0.28 |
| Somalia | 0.77 | 0.68 | 19.30 | 96.17 | 159.27 | 0.00 | 81.12 | 99.96 | 4.33 | 19.78 | 0.97 | 0.22 | 4.54 | 0.05 |
| South Africa | 1.47 | 1.07 | 30.85 | 172.18 | 221.61 | 8.84 | 80.25 | 73.87 | 23.52 | 39.91 | 5.94 | 0.24 | 14.92 | 0.55 |
| South Sudan | 1.23 | 1.04 | 30.36 | 97.09 | 151.15 | 1.01 | 50.04 | 87.71 | 12.71 | 22.64 | 1.01 | 0.26 | 5.16 | 0.25 |
| Spain | 5.23 | 2.40 | 61.22 | 230.52 | 235.06 | 15.18 | 25.64 | 7.29 | 19.55 | 25.55 | 5.14 | 0.06 | 24.35 | 0.65 |
| Sri Lanka | 5.05 | 2.83 | 69.41 | 186.31 | 258.79 | 3.32 | 74.25 | 87.79 | 7.99 | 28.06 | 1.44 | 0.18 | 9.65 | 0.50 |
| Sudan | 0.91 | 0.58 | 18.23 | 209.29 | 186.55 | 1.06 | 59.54 | 61.90 | 9.06 | 33.45 | 14.99 | 0.19 | 9.63 | 0.23 |
| Suriname | 2.90 | 1.96 | 61.22 | 171.12 | 305.29 | 7.80 | 53.10 | 75.43 | 16.93 | 19.18 | 4.84 | 0.24 | 11.52 | 0.50 |
| Sweden | 8.38 | 5.26 | 122.88 | 170.56 | 148.50 | 13.46 | 56.40 | 62.32 | 16.76 | 31.02 | 3.91 | 0.09 | 16.73 | 0.77 |
| Switzerland | 3.56 | 1.84 | 43.21 | 227.22 | 172.98 | 17.00 | 45.62 | 48.62 | 18.58 | 24.84 | 5.35 | 0.10 | 19.27 | 0.87 |
| Syrian Arab Republic | 0.78 | 0.45 | 12.61 | 278.72 | 224.53 | 1.07 | 31.40 | 21.81 | 19.76 | 25.66 | 7.91 | 0.18 | 16.91 | 0.37 |
| Taiwan (Province of China) | 2.58 | 0.99 | 25.72 | 254.98 | 208.76 | 7.99 | 45.83 | 44.71 | 12.04 | 22.76 | 2.03 | 0.35 | 16.43 | 0.67 |
| Tajikistan | 2.53 | 1.74 | 52.25 | 87.16 | 120.93 | 5.79 | 70.73 | 15.83 | 8.35 | 29.67 | 1.53 | 0.15 | 13.64 | 0.47 |
| Thailand | 1.41 | 0.74 | 20.82 | 207.05 | 181.65 | 7.53 | 51.05 | 83.28 | 7.50 | 18.08 | 1.88 | 0.31 | 14.78 | 0.51 |
| Timor-Leste | 0.87 | 0.58 | 17.66 | 142.07 | 152.25 | 2.14 | 87.74 | 95.51 | 3.14 | 31.70 | 1.78 | 0.19 | 12.09 | 0.27 |
| Togo | 0.88 | 0.68 | 21.33 | 140.36 | 118.56 | 4.27 | 95.55 | 92.50 | 6.73 | 30.51 | 2.29 | 0.27 | 6.79 | 0.27 |
| Tokelau | 1.13 | 0.66 | 17.98 | 157.76 | 311.90 | 3.93 | 66.09 | 91.55 | 17.88 | 14.80 | 3.99 | 0.26 | 11.73 | 0.43 |
| Tonga | 1.13 | 0.64 | 17.57 | 183.82 | 346.85 | 1.81 | 65.59 | 91.41 | 31.97 | 22.20 | 4.44 | 0.21 | 13.67 | 0.51 |
| Trinidad and Tobago | 4.81 | 3.06 | 93.20 | 180.96 | 485.61 | 7.42 | 69.25 | 84.06 | 27.32 | 25.53 | 6.26 | 0.28 | 11.22 | 0.62 |
| Tunisia | 1.36 | 0.78 | 19.93 | 257.84 | 226.12 | 2.22 | 43.64 | 31.89 | 18.82 | 24.19 | 2.22 | 0.21 | 15.60 | 0.43 |
| Turkey | 2.81 | 1.65 | 48.49 | 228.86 | 202.48 | 2.38 | 21.85 | 2.63 | 21.84 | 27.12 | 5.74 | 0.26 | 21.43 | 0.47 |
| Turkmenistan | 2.29 | 1.48 | 46.71 | 124.38 | 122.60 | 3.48 | 59.12 | 21.32 | 19.63 | 34.09 | 1.70 | 0.17 | 15.88 | 0.55 |
| Tuvalu | 1.26 | 0.75 | 22.14 | 154.17 | 311.50 | 2.87 | 71.42 | 94.11 | 15.03 | 14.36 | 3.92 | 0.27 | 12.27 | 0.43 |
| Uganda | 0.84 | 0.71 | 19.60 | 86.23 | 173.06 | 8.43 | 13.65 | 97.98 | 6.99 | 33.30 | 0.98 | 0.25 | 3.34 | 0.17 |
| Ukraine | 6.10 | 3.09 | 90.64 | 101.56 | 112.59 | 11.29 | 68.72 | 24.63 | 22.02 | 38.69 | 1.43 | 0.05 | 17.74 | 0.65 |
| United Arab Emirates | 4.25 | 2.79 | 75.46 | 344.25 | 397.24 | 4.37 | 14.28 | 2.78 | 33.53 | 36.77 | 15.42 | 0.31 | 10.28 | 0.62 |
| United Kingdom | 6.77 | 3.43 | 85.73 | 202.59 | 188.86 | 14.27 | 66.34 | 49.42 | 21.38 | 43.09 | 6.08 | 0.08 | 25.75 | 0.75 |
| United Republic of Tanzania | 1.12 | 0.93 | 26.22 | 103.45 | 115.66 | 6.32 | 70.73 | 88.36 | 10.43 | 21.60 | 0.98 | 0.35 | 8.44 | 0.26 |
| United States Virgin Islands | 4.67 | 2.92 | 83.61 | 193.74 | 265.79 | 7.03 | 36.61 | 68.59 | 30.39 | 24.29 | 2.94 | 0.32 | 6.06 | 0.67 |
| United States of America | 10.90 | 3.63 | 94.33 | 301.57 | 241.18 | 12.03 | 52.25 | 30.59 | 27.27 | 24.43 | 3.78 | 0.08 | 23.20 | 0.77 |
| Uruguay | 9.93 | 6.34 | 173.07 | 191.68 | 75.87 | 11.05 | 55.75 | 79.34 | 13.15 | 25.16 | 2.81 | 0.31 | 17.29 | 0.58 |
| Uzbekistan | 1.39 | 0.85 | 27.39 | 154.53 | 106.15 | 4.23 | 67.41 | 9.78 | 18.90 | 21.80 | 1.57 | 0.21 | 6.92 | 0.49 |
| Vanuatu | 0.93 | 0.62 | 16.67 | 141.33 | 282.19 | 2.86 | 44.86 | 84.90 | 14.63 | 32.70 | 2.38 | 0.25 | 9.19 | 0.36 |
| Venezuela (Bolivarian Republic of) | 4.00 | 2.63 | 77.16 | 244.83 | 277.16 | 9.21 | 38.68 | 82.15 | 18.75 | 35.40 | 1.24 | 0.32 | 12.47 | 0.51 |
| Viet Nam | 0.93 | 0.55 | 14.96 | 131.36 | 164.93 | 1.21 | 72.09 | 80.45 | 2.34 | 23.73 | 1.23 | 0.27 | 11.23 | 0.39 |
| Yemen | 0.78 | 0.52 | 15.00 | 207.26 | 145.90 | 1.52 | 72.97 | 77.48 | 6.21 | 20.16 | 6.29 | 0.15 | 13.68 | 0.18 |
| Zambia | 1.37 | 1.17 | 33.05 | 109.39 | 171.51 | 5.62 | 97.32 | 91.28 | 12.80 | 15.17 | 1.84 | 0.24 | 4.89 | 0.30 |
| Zimbabwe | 0.85 | 0.66 | 17.79 | 161.07 | 206.32 | 6.18 | 96.15 | 99.96 | 13.97 | 27.41 | 1.42 | 0.28 | 10.43 | 0.39 |
| ASIR: age standardized incidence rate; ASMR: age standardized mortality rate; ASDR: age standardized DALY rate; SDI: socio demographic index | | | | | | | | | | | | | | |

| **Table 2.** The values of age-standardized incidence rate, age-standardized mortality rate and age-standardized DALY rate of kidney cancer and its risk factors for each country in 2019 | | | | | | | | | | | | | | |
| --- | --- | --- | --- | --- | --- | --- | --- | --- | --- | --- | --- | --- | --- | --- |
| Country | ASIR | ASMR | ASDR | Chronic kidney disease | Diabetes mellitus type 2 | Alcohol use | Diet low in fruit | Diet low in vegetable | High body mass index | High systolic blood pressure | Lower physical activity | Occupation exposure to trichloroethylene | Smoking | SDI |
| Afghanistan | 1.51 | 0.98 | 27.25 | 385.22 | 412.01 | 0.21 | 70.62 | 98.11 | 12.76 | 22.89 | 7.01 | 0.19 | 5.59 | 0.34 |
| Albania | 4.58 | 2.32 | 57.83 | 202.71 | 161.15 | 7.93 | 20.25 | 3.10 | 26.20 | 42.26 | 2.28 | 0.05 | 16.42 | 0.68 |
| Algeria | 2.13 | 0.89 | 23.40 | 453.09 | 386.38 | 1.40 | 34.61 | 33.01 | 32.68 | 25.37 | 8.62 | 0.17 | 10.04 | 0.65 |
| American Samoa | 1.71 | 0.89 | 23.34 | 310.49 | 819.43 | 1.89 | 50.16 | 78.87 | 51.31 | 33.77 | 6.92 | 0.31 | 12.64 | 0.71 |
| Andorra | 8.85 | 3.68 | 84.00 | 230.97 | 212.86 | 16.90 | 36.02 | 26.06 | 29.06 | 34.00 | 6.37 | 0.08 | 16.33 | 0.89 |
| Angola | 1.43 | 1.11 | 30.18 | 126.40 | 253.23 | 8.89 | 56.35 | 69.45 | 13.11 | 36.01 | 3.63 | 0.40 | 5.22 | 0.47 |
| Antigua and Barbuda | 3.64 | 1.94 | 52.94 | 319.54 | 430.82 | 8.38 | 37.52 | 67.90 | 25.63 | 26.15 | 5.66 | 0.42 | 5.66 | 0.74 |
| Argentina | 9.06 | 5.18 | 130.01 | 271.61 | 262.98 | 13.51 | 28.22 | 45.32 | 23.17 | 23.27 | 0.90 | 0.37 | 14.20 | 0.71 |
| Armenia | 4.68 | 2.68 | 65.31 | 183.20 | 265.75 | 7.08 | 30.99 | 2.58 | 30.09 | 35.71 | 1.94 | 0.24 | 15.91 | 0.69 |
| Australia | 8.79 | 3.41 | 77.27 | 286.13 | 193.74 | 16.17 | 53.73 | 41.09 | 35.79 | 24.45 | 6.82 | 0.09 | 12.10 | 0.84 |
| Austria | 7.24 | 3.29 | 69.91 | 268.39 | 215.32 | 17.63 | 31.76 | 35.53 | 24.46 | 33.81 | 5.16 | 0.09 | 19.28 | 0.85 |
| Azerbaijan | 6.17 | 3.54 | 101.94 | 203.26 | 254.84 | 7.51 | 37.91 | 3.55 | 32.42 | 29.49 | 2.01 | 0.28 | 13.82 | 0.68 |
| Bahamas | 3.92 | 2.07 | 62.08 | 278.95 | 381.88 | 7.52 | 46.23 | 43.22 | 33.50 | 27.24 | 6.24 | 0.35 | 4.89 | 0.80 |
| Bahrain | 4.55 | 2.17 | 48.81 | 502.93 | 757.62 | 2.22 | 21.97 | 10.81 | 42.47 | 31.52 | 10.19 | 0.33 | 9.75 | 0.75 |
| Bangladesh | 0.86 | 0.59 | 15.62 | 143.47 | 223.64 | 0.42 | 85.68 | 88.41 | 7.79 | 26.04 | 2.20 | 0.30 | 8.98 | 0.48 |
| Barbados | 5.39 | 2.78 | 76.07 | 281.62 | 397.94 | 11.50 | 66.07 | 64.56 | 33.94 | 27.66 | 7.21 | 0.38 | 5.13 | 0.74 |
| Belarus | 11.17 | 4.50 | 120.65 | 143.85 | 126.90 | 16.83 | 55.59 | 8.42 | 25.92 | 42.66 | 1.80 | 0.08 | 16.77 | 0.75 |
| Belgium | 7.83 | 3.54 | 76.30 | 247.78 | 220.94 | 16.63 | 45.37 | 19.70 | 24.39 | 32.20 | 6.44 | 0.07 | 16.66 | 0.85 |
| Belize | 3.28 | 1.84 | 55.62 | 291.98 | 333.36 | 7.99 | 11.20 | 76.97 | 34.27 | 23.18 | 6.26 | 0.39 | 6.41 | 0.60 |
| Benin | 1.49 | 1.02 | 34.55 | 181.73 | 239.14 | 3.71 | 75.62 | 64.06 | 21.09 | 31.72 | 2.70 | 0.39 | 3.16 | 0.35 |
| Bermuda | 5.94 | 2.50 | 66.46 | 289.43 | 255.83 | 13.38 | 55.98 | 40.23 | 41.24 | 21.49 | 6.90 | 0.43 | 7.68 | 0.81 |
| Bhutan | 1.36 | 0.93 | 23.40 | 197.85 | 246.80 | 1.86 | 69.29 | 69.05 | 16.46 | 23.94 | 3.54 | 0.28 | 4.15 | 0.46 |
| Bolivia (Plurinational State of) | 3.11 | 2.25 | 54.61 | 257.65 | 265.30 | 7.17 | 49.47 | 81.96 | 28.24 | 14.54 | 2.65 | 0.47 | 3.90 | 0.57 |
| Bosnia and Herzegovina | 6.72 | 3.66 | 90.92 | 219.63 | 376.29 | 11.72 | 44.01 | 12.13 | 27.30 | 33.85 | 2.34 | 0.05 | 22.86 | 0.72 |
| Botswana | 2.35 | 1.52 | 41.15 | 237.42 | 338.31 | 6.60 | 89.63 | 82.62 | 26.88 | 36.81 | 3.60 | 0.18 | 7.79 | 0.63 |
| Brazil | 3.56 | 1.99 | 53.46 | 249.64 | 268.27 | 9.27 | 32.48 | 63.84 | 31.22 | 27.31 | 12.49 | 0.33 | 9.26 | 0.64 |
| Brunei Darussalam | 6.71 | 4.07 | 91.49 | 330.34 | 639.58 | 1.07 | 69.04 | 65.77 | 13.41 | 25.51 | 4.24 | 0.07 | 10.08 | 0.82 |
| Bulgaria | 5.94 | 3.05 | 86.24 | 232.62 | 253.87 | 16.95 | 59.23 | 4.83 | 28.54 | 31.07 | 2.24 | 0.08 | 22.46 | 0.76 |
| Burkina Faso | 1.24 | 0.84 | 29.36 | 166.83 | 220.98 | 8.33 | 99.24 | 93.78 | 14.71 | 23.33 | 1.47 | 0.19 | 3.01 | 0.26 |
| Burundi | 1.16 | 0.94 | 25.12 | 111.58 | 178.57 | 6.93 | 29.78 | 92.32 | 7.67 | 28.67 | 0.98 | 0.36 | 3.74 | 0.28 |
| Cabo Verde | 3.74 | 2.20 | 64.31 | 183.81 | 280.89 | 7.22 | 67.97 | 34.07 | 23.95 | 43.63 | 2.41 | 0.21 | 3.26 | 0.53 |
| Cambodia | 2.01 | 1.14 | 30.64 | 177.97 | 303.03 | 8.34 | 83.44 | 91.52 | 7.73 | 13.98 | 1.54 | 0.41 | 12.12 | 0.47 |
| Cameroon | 1.03 | 0.74 | 21.50 | 246.46 | 234.88 | 6.56 | 40.95 | 47.12 | 28.08 | 34.10 | 3.06 | 0.30 | 3.82 | 0.49 |
| Canada | 10.61 | 3.22 | 73.98 | 241.73 | 182.48 | 14.97 | 44.75 | 36.39 | 33.01 | 18.97 | 4.15 | 0.09 | 13.50 | 0.87 |
| Central African Republic | 0.93 | 0.80 | 22.83 | 113.90 | 276.68 | 4.40 | 71.98 | 99.96 | 7.54 | 32.69 | 3.41 | 0.24 | 3.41 | 0.27 |
| Chad | 0.94 | 0.69 | 22.79 | 158.73 | 223.36 | 4.00 | 93.44 | 99.95 | 8.67 | 25.14 | 1.93 | 0.22 | 4.04 | 0.24 |
| Chile | 8.48 | 4.47 | 104.31 | 315.57 | 311.62 | 13.60 | 49.22 | 27.69 | 34.06 | 26.84 | 3.51 | 0.38 | 14.92 | 0.76 |
| China | 3.21 | 1.27 | 34.28 | 161.52 | 201.06 | 8.59 | 45.26 | 3.34 | 13.20 | 28.04 | 2.31 | 0.36 | 14.52 | 0.69 |
| Colombia | 2.89 | 1.41 | 40.10 | 308.32 | 325.85 | 7.35 | 37.03 | 64.64 | 24.99 | 20.98 | 2.85 | 0.44 | 5.36 | 0.63 |
| Comoros | 1.63 | 1.28 | 33.39 | 129.09 | 165.96 | 0.87 | 45.46 | 99.91 | 19.16 | 30.49 | 0.82 | 0.34 | 4.77 | 0.46 |
| Congo | 1.86 | 1.46 | 37.90 | 141.29 | 282.89 | 7.51 | 62.56 | 97.98 | 20.84 | 37.96 | 4.30 | 0.27 | 4.32 | 0.57 |
| Cook Islands | 1.88 | 0.70 | 19.56 | 277.12 | 591.63 | 11.56 | 42.22 | 71.93 | 50.32 | 28.25 | 6.19 | 0.38 | 10.41 | 0.76 |
| Costa Rica | 4.37 | 2.19 | 56.43 | 454.86 | 348.37 | 8.24 | 34.80 | 73.56 | 38.11 | 42.67 | 1.51 | 0.35 | 6.81 | 0.68 |
| Croatia | 9.96 | 4.47 | 103.57 | 253.28 | 295.89 | 14.01 | 41.57 | 40.18 | 29.66 | 37.64 | 2.34 | 0.07 | 19.82 | 0.79 |
| Cuba | 4.19 | 1.97 | 53.87 | 245.73 | 337.19 | 8.97 | 33.89 | 29.99 | 33.64 | 19.05 | 6.49 | 0.36 | 13.64 | 0.67 |
| Cyprus | 4.67 | 2.04 | 46.04 | 285.71 | 337.29 | 14.86 | 41.18 | 44.67 | 20.11 | 32.49 | 4.06 | 0.07 | 19.04 | 0.84 |
| Czechia | 15.66 | 6.42 | 144.85 | 213.16 | 445.30 | 19.93 | 58.20 | 48.19 | 29.68 | 29.03 | 3.00 | 0.09 | 16.45 | 0.83 |
| Côte d'Ivoire | 1.49 | 1.07 | 33.77 | 193.77 | 241.05 | 7.21 | 52.23 | 77.84 | 19.10 | 39.81 | 2.81 | 0.27 | 6.11 | 0.41 |
| Democratic People's Republic of Korea | 2.18 | 0.93 | 28.50 | 186.21 | 198.95 | 6.96 | 55.78 | 36.89 | 4.53 | 16.90 | 1.83 | 0.35 | 12.43 | 0.56 |
| Democratic Republic of the Congo | 0.92 | 0.74 | 19.77 | 123.39 | 254.34 | 3.71 | 72.06 | 99.96 | 9.92 | 27.90 | 3.67 | 0.31 | 3.14 | 0.38 |
| Denmark | 8.35 | 4.00 | 90.52 | 238.75 | 193.21 | 14.89 | 34.18 | 30.18 | 24.80 | 34.64 | 4.82 | 0.09 | 16.37 | 0.89 |
| Djibouti | 2.16 | 1.65 | 43.25 | 133.33 | 193.78 | 1.00 | 91.33 | 71.33 | 15.08 | 31.39 | 1.04 | 0.23 | 8.03 | 0.46 |
| Dominica | 4.25 | 2.52 | 72.89 | 309.60 | 496.08 | 9.34 | 4.40 | 51.06 | 36.76 | 26.83 | 5.53 | 0.32 | 5.63 | 0.73 |
| Dominican Republic | 4.33 | 2.40 | 72.84 | 229.39 | 223.74 | 9.03 | 13.95 | 60.63 | 26.71 | 25.59 | 8.65 | 0.41 | 8.05 | 0.59 |
| Ecuador | 3.42 | 2.06 | 52.63 | 341.85 | 305.67 | 6.12 | 21.82 | 65.79 | 37.18 | 15.51 | 3.20 | 0.47 | 4.78 | 0.64 |
| Egypt | 2.23 | 1.04 | 29.26 | 473.45 | 295.24 | 0.57 | 23.41 | 2.58 | 42.12 | 30.15 | 8.78 | 0.22 | 13.20 | 0.66 |
| El Salvador | 2.62 | 1.51 | 38.95 | 384.65 | 366.85 | 5.74 | 53.72 | 62.72 | 28.23 | 28.94 | 1.41 | 0.41 | 4.71 | 0.57 |
| Equatorial Guinea | 2.27 | 1.69 | 41.93 | 156.63 | 283.09 | 8.84 | 35.98 | 87.09 | 38.07 | 41.63 | 4.83 | 0.25 | 4.17 | 0.69 |
| Eritrea | 1.50 | 1.21 | 32.41 | 119.96 | 195.24 | 2.58 | 70.82 | 94.59 | 8.44 | 20.97 | 1.05 | 0.34 | 3.37 | 0.40 |
| Estonia | 12.96 | 5.63 | 128.50 | 206.13 | 179.11 | 19.95 | 58.55 | 30.00 | 34.69 | 35.68 | 1.81 | 0.08 | 17.38 | 0.84 |
| Eswatini | 2.44 | 1.80 | 49.97 | 240.58 | 368.09 | 6.13 | 67.77 | 93.80 | 33.11 | 34.35 | 4.04 | 0.16 | 2.61 | 0.58 |
| Ethiopia | 1.54 | 1.26 | 30.07 | 116.15 | 148.03 | 4.33 | 97.16 | 92.30 | 10.52 | 20.41 | 1.09 | 0.39 | 1.71 | 0.34 |
| Fiji | 1.48 | 0.82 | 22.67 | 260.00 | 797.04 | 3.96 | 81.49 | 73.44 | 36.67 | 37.36 | 5.00 | 0.28 | 9.42 | 0.66 |
| Finland | 10.32 | 3.99 | 86.18 | 190.57 | 287.80 | 15.16 | 57.43 | 46.38 | 26.19 | 36.51 | 4.81 | 0.08 | 12.97 | 0.86 |
| France | 8.79 | 3.77 | 85.18 | 229.07 | 140.55 | 16.68 | 56.94 | 48.92 | 24.90 | 28.82 | 7.94 | 0.07 | 15.28 | 0.83 |
| Gabon | 2.89 | 2.10 | 55.40 | 178.38 | 320.22 | 9.85 | 24.86 | 85.07 | 29.74 | 31.93 | 3.66 | 0.21 | 4.05 | 0.66 |
| Gambia | 1.14 | 0.86 | 23.71 | 177.72 | 213.80 | 3.94 | 99.83 | 91.77 | 17.31 | 37.81 | 2.39 | 0.27 | 5.61 | 0.40 |
| Georgia | 5.41 | 2.97 | 85.27 | 176.68 | 305.48 | 9.53 | 61.06 | 37.95 | 31.18 | 36.87 | 1.90 | 0.26 | 14.95 | 0.70 |
| Germany | 10.44 | 4.04 | 87.08 | 266.56 | 340.97 | 19.37 | 49.83 | 43.08 | 28.61 | 30.01 | 3.72 | 0.09 | 16.35 | 0.90 |
| Ghana | 1.50 | 1.00 | 30.71 | 175.56 | 260.66 | 5.55 | 24.64 | 71.87 | 24.05 | 30.84 | 2.60 | 0.37 | 2.26 | 0.56 |
| Greece | 7.29 | 3.19 | 72.99 | 267.10 | 227.35 | 13.53 | 19.06 | 3.73 | 27.75 | 22.77 | 2.99 | 0.06 | 23.53 | 0.79 |
| Greenland | 11.52 | 6.31 | 148.46 | 228.09 | 200.43 | 11.41 | 40.75 | 22.79 | 33.68 | 19.40 | 3.50 | 0.09 | 21.21 | 0.76 |
| Grenada | 3.45 | 1.99 | 55.83 | 335.34 | 481.18 | 10.79 | 34.84 | 83.65 | 27.16 | 24.86 | 5.18 | 0.39 | 5.45 | 0.67 |
| Guam | 3.15 | 1.33 | 38.57 | 249.06 | 349.01 | 4.53 | 36.22 | 64.13 | 48.59 | 31.54 | 4.36 | 0.26 | 10.39 | 0.81 |
| Guatemala | 2.51 | 1.61 | 43.93 | 381.49 | 437.86 | 4.30 | 54.08 | 64.68 | 19.49 | 30.01 | 0.67 | 0.38 | 4.28 | 0.53 |
| Guinea | 1.11 | 0.82 | 26.41 | 174.56 | 214.25 | 1.63 | 45.29 | 80.24 | 13.47 | 30.40 | 1.94 | 0.20 | 6.39 | 0.33 |
| Guinea-Bissau | 1.29 | 0.96 | 29.64 | 177.10 | 235.42 | 4.54 | 66.78 | 98.92 | 11.96 | 33.65 | 2.41 | 0.25 | 2.33 | 0.36 |
| Guyana | 3.40 | 2.06 | 63.93 | 284.74 | 533.58 | 8.58 | 63.70 | 64.15 | 25.97 | 23.76 | 5.77 | 0.32 | 6.15 | 0.62 |
| Haiti | 1.74 | 1.22 | 39.60 | 221.80 | 429.87 | 6.86 | 54.32 | 97.47 | 9.11 | 38.37 | 4.36 | 0.28 | 3.30 | 0.43 |
| Honduras | 2.48 | 1.75 | 42.02 | 325.82 | 380.91 | 4.39 | 50.69 | 68.70 | 19.88 | 28.56 | 1.19 | 0.42 | 6.66 | 0.50 |
| Hungary | 9.89 | 4.84 | 117.51 | 221.15 | 298.35 | 14.90 | 56.51 | 17.23 | 32.99 | 28.25 | 2.04 | 0.08 | 18.74 | 0.79 |
| Iceland | 12.54 | 4.96 | 113.24 | 194.95 | 219.96 | 12.83 | 52.43 | 51.92 | 31.03 | 25.84 | 6.48 | 0.10 | 13.32 | 0.87 |
| India | 1.11 | 0.77 | 20.01 | 183.62 | 309.20 | 4.17 | 96.13 | 60.32 | 11.78 | 24.22 | 2.21 | 0.25 | 5.88 | 0.57 |
| Indonesia | 2.49 | 1.40 | 37.60 | 176.90 | 236.88 | 0.82 | 58.20 | 69.29 | 16.03 | 39.03 | 3.11 | 0.37 | 12.42 | 0.66 |
| Iran (Islamic Republic of) | 3.27 | 1.33 | 34.36 | 420.53 | 323.31 | 0.92 | 17.08 | 7.52 | 30.57 | 25.20 | 6.93 | 0.20 | 8.72 | 0.67 |
| Iraq | 3.96 | 1.84 | 49.21 | 484.35 | 424.91 | 1.01 | 54.65 | 12.63 | 32.50 | 39.12 | 9.05 | 0.20 | 12.47 | 0.67 |
| Ireland | 9.14 | 3.73 | 84.36 | 252.62 | 221.61 | 15.54 | 46.74 | 29.01 | 30.43 | 32.17 | 9.50 | 0.08 | 14.19 | 0.87 |
| Israel | 6.27 | 3.07 | 65.70 | 299.29 | 258.62 | 5.61 | 21.11 | 2.81 | 27.40 | 31.95 | 6.23 | 0.08 | 12.98 | 0.80 |
| Italy | 10.99 | 3.42 | 78.03 | 218.16 | 289.31 | 14.49 | 25.73 | 25.55 | 22.98 | 26.09 | 6.51 | 0.07 | 14.57 | 0.80 |
| Jamaica | 2.34 | 1.22 | 35.91 | 267.60 | 446.09 | 6.78 | 43.26 | 55.70 | 32.32 | 24.84 | 7.21 | 0.38 | 7.97 | 0.68 |
| Japan | 4.54 | 2.03 | 43.46 | 291.45 | 168.41 | 15.23 | 69.50 | 15.56 | 10.71 | 25.78 | 4.36 | 0.10 | 13.60 | 0.87 |
| Jordan | 3.12 | 1.38 | 34.19 | 494.06 | 395.59 | 1.09 | 49.91 | 32.20 | 42.74 | 27.65 | 6.52 | 0.16 | 17.25 | 0.73 |
| Kazakhstan | 6.15 | 3.34 | 90.06 | 183.31 | 287.69 | 8.66 | 56.73 | 4.86 | 34.23 | 43.52 | 2.80 | 0.32 | 12.31 | 0.72 |
| Kenya | 0.97 | 0.79 | 19.70 | 117.67 | 161.74 | 5.18 | 64.73 | 67.53 | 19.45 | 33.13 | 0.95 | 0.45 | 4.01 | 0.51 |
| Kiribati | 3.89 | 2.54 | 75.58 | 211.56 | 610.54 | 2.16 | 57.68 | 82.31 | 24.11 | 17.69 | 4.68 | 0.21 | 19.20 | 0.53 |
| Kuwait | 3.99 | 1.49 | 37.57 | 449.98 | 495.49 | 0.19 | 43.88 | 5.78 | 53.49 | 24.36 | 12.60 | 0.30 | 12.20 | 0.85 |
| Kyrgyzstan | 3.26 | 1.89 | 53.04 | 154.85 | 151.50 | 6.44 | 67.17 | 9.87 | 21.84 | 17.20 | 1.80 | 0.28 | 13.33 | 0.60 |
| Lao People's Democratic Republic | 1.84 | 1.12 | 31.10 | 231.15 | 340.57 | 8.84 | 49.92 | 60.40 | 11.55 | 23.36 | 1.58 | 0.40 | 11.36 | 0.49 |
| Latvia | 12.09 | 5.17 | 125.34 | 167.47 | 181.39 | 18.62 | 69.75 | 28.94 | 30.56 | 44.03 | 2.96 | 0.07 | 16.07 | 0.82 |
| Lebanon | 5.50 | 2.41 | 56.61 | 487.66 | 383.50 | 1.73 | 19.54 | 2.77 | 35.33 | 34.52 | 9.47 | 0.22 | 18.92 | 0.71 |
| Lesotho | 1.66 | 1.29 | 35.65 | 203.54 | 300.61 | 6.99 | 90.38 | 92.15 | 24.32 | 27.20 | 2.45 | 0.18 | 8.52 | 0.51 |
| Liberia | 1.26 | 0.92 | 27.15 | 176.22 | 272.79 | 6.23 | 70.64 | 93.38 | 22.28 | 35.52 | 3.10 | 0.32 | 3.48 | 0.37 |
| Libya | 4.20 | 2.23 | 55.41 | 451.25 | 454.79 | 0.55 | 38.06 | 31.44 | 39.04 | 40.28 | 9.48 | 0.18 | 11.47 | 0.71 |
| Lithuania | 11.70 | 5.41 | 134.17 | 150.96 | 141.66 | 18.91 | 59.51 | 27.12 | 27.23 | 47.44 | 3.10 | 0.07 | 13.57 | 0.84 |
| Luxembourg | 4.02 | 1.75 | 39.27 | 258.74 | 339.16 | 16.98 | 49.60 | 32.79 | 22.75 | 33.87 | 4.84 | 0.06 | 15.31 | 0.90 |
| Madagascar | 1.12 | 0.90 | 23.83 | 106.12 | 168.92 | 2.97 | 71.10 | 98.01 | 10.73 | 30.59 | 1.01 | 0.32 | 3.50 | 0.40 |
| Malawi | 3.07 | 2.40 | 64.44 | 125.19 | 216.58 | 4.02 | 61.03 | 91.19 | 16.65 | 43.30 | 1.18 | 0.41 | 4.45 | 0.38 |
| Malaysia | 3.49 | 1.63 | 39.12 | 264.75 | 334.02 | 2.33 | 46.32 | 60.96 | 25.81 | 38.87 | 4.93 | 0.39 | 10.29 | 0.74 |
| Maldives | 2.61 | 1.09 | 26.59 | 296.69 | 271.15 | 2.80 | 60.04 | 47.97 | 14.88 | 29.98 | 4.17 | 0.36 | 13.25 | 0.56 |
| Mali | 1.20 | 0.83 | 28.15 | 180.40 | 208.96 | 1.34 | 72.34 | 88.29 | 12.68 | 22.00 | 1.85 | 0.24 | 4.17 | 0.26 |
| Malta | 6.85 | 2.95 | 67.88 | 263.03 | 314.06 | 13.17 | 48.34 | 8.95 | 23.08 | 37.16 | 12.03 | 0.08 | 15.80 | 0.80 |
| Marshall Islands | 1.48 | 0.90 | 25.30 | 239.75 | 795.18 | 4.10 | 64.82 | 89.82 | 21.75 | 17.59 | 5.63 | 0.19 | 9.07 | 0.54 |
| Mauritania | 1.39 | 0.99 | 26.83 | 202.79 | 168.02 | 0.00 | 94.15 | 91.35 | 26.79 | 31.72 | 10.16 | 0.18 | 5.96 | 0.50 |
| Mauritius | 3.38 | 1.35 | 37.71 | 390.50 | 591.43 | 7.41 | 76.05 | 52.66 | 24.65 | 25.02 | 3.75 | 0.35 | 8.04 | 0.71 |
| Mexico | 5.26 | 2.97 | 77.55 | 469.06 | 477.63 | 8.62 | 37.61 | 56.07 | 36.30 | 28.66 | 3.69 | 0.41 | 5.07 | 0.65 |
| Micronesia (Federated States of) | 1.90 | 1.07 | 30.23 | 293.81 | 580.29 | 3.98 | 65.56 | 90.42 | 31.18 | 16.46 | 6.08 | 0.30 | 14.46 | 0.58 |
| Monaco | 10.61 | 4.27 | 98.25 | 227.19 | 209.58 | 11.08 | 16.69 | 4.05 | 36.16 | 34.92 | 7.48 | 0.07 | 16.30 | 0.90 |
| Mongolia | 3.75 | 2.58 | 63.28 | 194.46 | 104.83 | 8.70 | 94.94 | 70.13 | 24.41 | 36.97 | 1.73 | 0.28 | 10.91 | 0.61 |
| Montenegro | 6.68 | 3.30 | 81.22 | 265.58 | 335.34 | 13.16 | 20.32 | 2.97 | 34.72 | 38.07 | 2.59 | 0.06 | 24.57 | 0.79 |
| Morocco | 1.71 | 0.86 | 22.69 | 436.35 | 345.08 | 0.93 | 22.75 | 17.24 | 29.55 | 39.19 | 8.40 | 0.23 | 7.29 | 0.55 |
| Mozambique | 1.25 | 0.97 | 27.68 | 120.34 | 210.50 | 3.18 | 83.97 | 94.48 | 16.17 | 43.65 | 1.01 | 0.32 | 3.98 | 0.31 |
| Myanmar | 2.08 | 1.16 | 33.05 | 227.92 | 341.32 | 4.56 | 74.17 | 56.66 | 11.22 | 26.62 | 1.86 | 0.31 | 8.45 | 0.52 |
| Namibia | 2.41 | 1.73 | 43.83 | 205.43 | 266.09 | 9.66 | 84.56 | 81.86 | 20.78 | 31.80 | 5.78 | 0.15 | 4.76 | 0.61 |
| Nauru | 2.90 | 1.39 | 41.23 | 270.60 | 517.47 | 6.74 | 54.63 | 82.92 | 32.83 | 37.07 | 4.76 | 0.34 | 13.76 | 0.62 |
| Nepal | 1.05 | 0.79 | 19.47 | 213.40 | 250.46 | 4.13 | 67.63 | 64.83 | 11.22 | 19.24 | 2.33 | 0.34 | 7.55 | 0.42 |
| Netherlands | 10.33 | 4.35 | 95.18 | 236.91 | 182.06 | 14.46 | 43.98 | 49.34 | 22.78 | 30.26 | 2.12 | 0.09 | 15.55 | 0.88 |
| New Zealand | 9.95 | 3.40 | 81.11 | 286.18 | 173.84 | 15.08 | 43.66 | 38.19 | 34.77 | 23.16 | 5.96 | 0.09 | 10.88 | 0.84 |
| Nicaragua | 3.05 | 1.79 | 45.79 | 428.19 | 377.88 | 5.62 | 83.29 | 96.60 | 24.33 | 33.25 | 1.28 | 0.42 | 5.25 | 0.52 |
| Niger | 0.74 | 0.55 | 16.95 | 158.33 | 159.06 | 0.81 | 86.69 | 59.30 | 9.34 | 30.10 | 2.35 | 0.25 | 2.60 | 0.16 |
| Nigeria | 1.15 | 0.83 | 25.31 | 180.05 | 163.70 | 5.24 | 58.65 | 54.93 | 19.18 | 43.17 | 3.00 | 0.22 | 2.17 | 0.52 |
| Niue | 3.15 | 1.26 | 37.35 | 291.46 | 703.98 | 6.05 | 52.74 | 81.30 | 41.97 | 32.30 | 5.40 | 0.29 | 9.34 | 0.71 |
| North Macedonia | 4.36 | 2.21 | 57.50 | 259.42 | 384.21 | 10.73 | 35.83 | 3.65 | 29.20 | 35.21 | 2.32 | 0.06 | 21.75 | 0.74 |
| Northern Mariana Islands | 3.93 | 1.60 | 43.18 | 310.60 | 468.47 | 3.80 | 42.98 | 72.63 | 47.78 | 29.41 | 5.70 | 0.40 | 10.75 | 0.77 |
| Norway | 10.33 | 4.02 | 87.63 | 201.39 | 246.87 | 13.02 | 39.42 | 52.56 | 22.60 | 36.17 | 5.76 | 0.08 | 11.81 | 0.91 |
| Oman | 3.24 | 1.40 | 33.00 | 442.90 | 410.33 | 0.96 | 20.64 | 26.75 | 42.32 | 34.56 | 9.89 | 0.23 | 6.15 | 0.78 |
| Pakistan | 1.57 | 1.16 | 30.95 | 193.28 | 302.59 | 0.99 | 73.43 | 85.79 | 12.44 | 34.79 | 3.78 | 0.25 | 7.48 | 0.45 |
| Palau | 1.77 | 0.71 | 20.59 | 313.37 | 607.88 | 5.76 | 50.58 | 79.83 | 42.51 | 27.86 | 5.16 | 0.32 | 8.89 | 0.74 |
| Palestine | 3.27 | 1.59 | 40.30 | 474.96 | 452.69 | 1.75 | 49.02 | 27.72 | 26.54 | 21.50 | 7.48 | 0.17 | 12.60 | 0.59 |
| Panama | 3.76 | 1.97 | 52.40 | 350.46 | 380.48 | 8.96 | 53.46 | 78.67 | 24.91 | 28.80 | 1.24 | 0.37 | 4.13 | 0.69 |
| Papua New Guinea | 0.79 | 0.49 | 14.81 | 122.58 | 463.54 | 2.74 | 64.81 | 76.50 | 12.79 | 15.29 | 3.74 | 0.24 | 9.21 | 0.39 |
| Paraguay | 2.99 | 1.76 | 46.56 | 300.45 | 283.64 | 11.15 | 37.58 | 74.19 | 28.89 | 29.23 | 2.55 | 0.41 | 10.00 | 0.64 |
| Peru | 3.68 | 2.13 | 53.83 | 271.47 | 193.60 | 9.19 | 45.86 | 66.84 | 31.01 | 19.59 | 3.07 | 0.53 | 2.67 | 0.65 |
| Philippines | 2.62 | 1.29 | 38.95 | 267.29 | 248.84 | 8.92 | 44.20 | 62.70 | 12.71 | 21.51 | 1.06 | 0.37 | 12.31 | 0.62 |
| Poland | 9.10 | 5.33 | 125.85 | 207.93 | 280.66 | 16.34 | 58.30 | 9.92 | 29.78 | 33.36 | 3.68 | 0.07 | 16.78 | 0.80 |
| Portugal | 5.79 | 2.14 | 51.95 | 230.21 | 333.22 | 15.41 | 38.57 | 9.98 | 22.94 | 27.74 | 7.39 | 0.08 | 12.91 | 0.74 |
| Puerto Rico | 4.12 | 1.85 | 48.42 | 328.27 | 461.36 | 10.13 | 32.36 | 84.29 | 46.94 | 28.83 | 7.65 | 0.05 | 7.31 | 0.81 |
| Qatar | 6.94 | 4.07 | 72.83 | 506.35 | 818.03 | 1.93 | 12.10 | 3.17 | 56.52 | 29.06 | 11.57 | 0.32 | 10.12 | 0.83 |
| Republic of Korea | 4.09 | 1.80 | 40.85 | 238.61 | 242.40 | 17.98 | 41.05 | 10.93 | 17.44 | 14.97 | 4.05 | 0.09 | 13.53 | 0.88 |
| Republic of Moldova | 6.24 | 2.70 | 83.54 | 123.42 | 197.43 | 16.09 | 60.83 | 40.78 | 25.95 | 44.43 | 1.78 | 0.04 | 16.15 | 0.70 |
| Romania | 6.68 | 3.17 | 88.13 | 203.26 | 197.04 | 14.83 | 46.29 | 2.61 | 32.61 | 39.03 | 3.27 | 0.07 | 15.73 | 0.76 |
| Russian Federation | 10.00 | 4.35 | 112.81 | 202.71 | 141.06 | 15.55 | 58.55 | 36.39 | 30.82 | 37.91 | 1.76 | 0.07 | 18.09 | 0.81 |
| Rwanda | 1.65 | 1.27 | 32.84 | 125.80 | 172.39 | 9.35 | 2.29 | 84.33 | 14.55 | 23.90 | 1.01 | 0.28 | 6.33 | 0.43 |
| Saint Kitts and Nevis | 5.44 | 2.85 | 77.10 | 374.69 | 429.24 | 5.09 | 76.49 | 69.05 | 32.00 | 32.91 | 6.05 | 0.37 | 4.31 | 0.75 |
| Saint Lucia | 2.98 | 1.61 | 46.64 | 300.45 | 546.46 | 11.84 | 45.13 | 82.55 | 28.90 | 27.10 | 5.89 | 0.36 | 6.50 | 0.67 |
| Saint Vincent and the Grenadines | 3.03 | 1.74 | 50.05 | 272.42 | 505.89 | 10.56 | 32.68 | 76.28 | 29.18 | 24.82 | 5.84 | 0.32 | 5.63 | 0.63 |
| Samoa | 1.74 | 0.93 | 23.56 | 265.28 | 513.87 | 4.85 | 60.37 | 97.52 | 35.18 | 25.21 | 5.52 | 0.19 | 14.96 | 0.64 |
| San Marino | 5.45 | 2.31 | 52.26 | 202.29 | 231.43 | 14.43 | 38.16 | 29.54 | 31.62 | 33.23 | 6.56 | 0.08 | 14.09 | 0.88 |
| Sao Tome and Principe | 1.68 | 1.07 | 34.73 | 238.22 | 232.75 | 6.97 | 27.66 | 86.66 | 22.80 | 45.69 | 2.50 | 0.17 | 2.87 | 0.50 |
| Saudi Arabia | 3.96 | 1.60 | 40.64 | 561.38 | 462.07 | 0.60 | 44.85 | 38.96 | 49.96 | 25.36 | 17.25 | 0.22 | 9.07 | 0.81 |
| Senegal | 1.30 | 0.95 | 27.72 | 169.79 | 297.24 | 0.85 | 81.13 | 69.94 | 16.41 | 32.76 | 3.78 | 0.21 | 4.63 | 0.39 |
| Serbia | 7.18 | 3.91 | 96.30 | 238.04 | 336.63 | 13.17 | 31.75 | 30.12 | 29.41 | 40.20 | 2.30 | 0.06 | 21.14 | 0.77 |
| Seychelles | 4.01 | 1.89 | 48.18 | 319.29 | 504.42 | 11.11 | 51.73 | 80.18 | 22.94 | 30.82 | 2.39 | 0.41 | 10.52 | 0.72 |
| Sierra Leone | 1.23 | 0.83 | 29.42 | 165.17 | 154.37 | 5.06 | 74.19 | 80.96 | 12.53 | 45.68 | 2.47 | 0.25 | 6.28 | 0.35 |
| Singapore | 4.55 | 2.04 | 45.93 | 288.74 | 258.76 | 5.72 | 32.51 | 18.43 | 18.30 | 14.57 | 3.90 | 0.09 | 7.50 | 0.86 |
| Slovakia | 10.79 | 5.12 | 123.08 | 225.24 | 230.08 | 16.37 | 60.54 | 31.08 | 27.10 | 37.04 | 2.69 | 0.08 | 14.05 | 0.81 |
| Slovenia | 10.33 | 3.99 | 91.89 | 212.04 | 232.73 | 11.32 | 36.31 | 42.91 | 31.87 | 39.48 | 2.68 | 0.08 | 18.58 | 0.84 |
| Solomon Islands | 1.47 | 0.83 | 24.60 | 185.61 | 493.92 | 3.01 | 66.40 | 99.96 | 18.97 | 14.11 | 3.82 | 0.40 | 12.68 | 0.41 |
| Somalia | 0.93 | 0.79 | 21.73 | 113.50 | 194.07 | 0.00 | 87.95 | 99.96 | 4.54 | 26.32 | 0.96 | 0.27 | 3.87 | 0.08 |
| South Africa | 1.94 | 1.32 | 33.93 | 238.60 | 329.64 | 10.29 | 79.84 | 69.88 | 35.50 | 39.53 | 5.68 | 0.14 | 7.43 | 0.68 |
| South Sudan | 1.51 | 1.26 | 33.42 | 116.04 | 183.25 | 0.79 | 50.29 | 85.09 | 20.34 | 30.72 | 1.07 | 0.30 | 4.12 | 0.36 |
| Spain | 8.22 | 3.03 | 71.64 | 228.28 | 288.93 | 15.19 | 26.22 | 23.22 | 27.59 | 30.33 | 5.87 | 0.07 | 19.44 | 0.77 |
| Sri Lanka | 6.24 | 2.60 | 64.74 | 287.43 | 514.72 | 7.54 | 74.02 | 69.14 | 16.41 | 29.44 | 1.51 | 0.31 | 5.36 | 0.69 |
| Sudan | 2.22 | 1.21 | 33.39 | 374.99 | 359.63 | 0.07 | 52.26 | 51.43 | 24.65 | 39.84 | 16.72 | 0.25 | 9.00 | 0.52 |
| Suriname | 2.94 | 1.75 | 52.54 | 319.13 | 491.46 | 8.22 | 49.60 | 72.42 | 27.45 | 20.03 | 6.11 | 0.33 | 10.33 | 0.64 |
| Sweden | 7.04 | 3.97 | 83.56 | 172.22 | 221.49 | 14.32 | 45.71 | 46.55 | 26.33 | 32.22 | 3.78 | 0.09 | 11.85 | 0.87 |
| Switzerland | 6.25 | 2.70 | 57.72 | 250.68 | 226.79 | 16.09 | 45.58 | 43.94 | 23.36 | 19.64 | 5.82 | 0.10 | 14.75 | 0.93 |
| Syrian Arab Republic | 1.54 | 0.66 | 17.66 | 442.35 | 345.74 | 0.69 | 39.10 | 43.96 | 30.66 | 27.74 | 8.56 | 0.18 | 12.55 | 0.62 |
| Taiwan (Province of China) | 10.12 | 3.19 | 75.44 | 309.16 | 259.23 | 8.47 | 31.44 | 15.79 | 20.65 | 20.06 | 2.50 | 0.42 | 11.19 | 0.87 |
| Tajikistan | 3.38 | 2.26 | 61.65 | 141.89 | 268.25 | 4.18 | 69.44 | 9.56 | 12.19 | 29.94 | 1.61 | 0.17 | 7.96 | 0.54 |
| Thailand | 2.29 | 0.89 | 24.30 | 304.63 | 256.32 | 10.35 | 32.67 | 69.97 | 19.83 | 17.72 | 2.31 | 0.40 | 9.25 | 0.69 |
| Timor-Leste | 1.63 | 0.98 | 26.38 | 204.73 | 275.66 | 4.55 | 88.97 | 91.72 | 6.28 | 33.81 | 1.68 | 0.30 | 11.13 | 0.51 |
| Togo | 1.22 | 0.86 | 26.19 | 179.89 | 167.33 | 3.67 | 96.68 | 95.40 | 15.24 | 30.98 | 2.51 | 0.28 | 4.66 | 0.42 |
| Tokelau | 1.75 | 0.85 | 23.00 | 247.56 | 505.30 | 5.63 | 57.57 | 85.27 | 33.26 | 25.78 | 4.68 | 0.29 | 10.86 | 0.63 |
| Tonga | 1.64 | 0.87 | 24.05 | 253.75 | 501.81 | 2.77 | 61.09 | 87.52 | 43.32 | 33.29 | 5.13 | 0.25 | 11.30 | 0.64 |
| Trinidad and Tobago | 3.35 | 1.75 | 53.82 | 287.21 | 554.52 | 9.33 | 63.80 | 77.33 | 41.15 | 37.47 | 7.69 | 0.36 | 8.08 | 0.76 |
| Tunisia | 2.77 | 1.20 | 29.27 | 452.53 | 408.64 | 3.62 | 32.57 | 8.07 | 33.15 | 27.23 | 2.77 | 0.20 | 13.28 | 0.67 |
| Turkey | 4.99 | 1.96 | 52.75 | 432.17 | 273.83 | 2.79 | 12.53 | 2.56 | 36.70 | 26.12 | 6.98 | 0.27 | 16.81 | 0.75 |
| Turkmenistan | 6.24 | 3.43 | 105.97 | 184.40 | 217.05 | 7.05 | 42.94 | 2.99 | 36.42 | 36.11 | 2.19 | 0.23 | 10.54 | 0.67 |
| Tuvalu | 1.63 | 0.88 | 24.64 | 239.04 | 528.09 | 3.78 | 64.90 | 89.85 | 32.15 | 24.37 | 4.25 | 0.29 | 11.83 | 0.59 |
| Uganda | 2.05 | 1.59 | 41.76 | 110.28 | 222.43 | 9.64 | 21.33 | 91.95 | 15.10 | 29.57 | 1.02 | 0.31 | 3.11 | 0.40 |
| Ukraine | 9.91 | 4.34 | 128.36 | 128.03 | 144.46 | 16.11 | 69.00 | 11.17 | 24.69 | 36.29 | 1.37 | 0.06 | 15.60 | 0.74 |
| United Arab Emirates | 8.44 | 4.72 | 123.66 | 516.47 | 589.90 | 3.79 | 30.54 | 31.41 | 53.64 | 27.25 | 16.61 | 0.33 | 9.98 | 0.88 |
| United Kingdom | 9.65 | 3.93 | 88.59 | 215.62 | 361.33 | 15.20 | 58.56 | 43.02 | 30.14 | 25.37 | 6.92 | 0.08 | 16.38 | 0.85 |
| United Republic of Tanzania | 1.99 | 1.51 | 40.60 | 130.52 | 167.46 | 6.38 | 51.98 | 81.23 | 18.45 | 45.96 | 1.03 | 0.39 | 7.66 | 0.42 |
| United States Virgin Islands | 6.92 | 3.79 | 102.85 | 296.73 | 403.20 | 9.27 | 26.22 | 57.69 | 48.61 | 27.99 | 3.44 | 0.36 | 5.16 | 0.80 |
| United States of America | 11.85 | 3.52 | 84.02 | 318.61 | 360.83 | 13.61 | 45.68 | 38.04 | 41.92 | 18.90 | 3.80 | 0.09 | 14.51 | 0.86 |
| Uruguay | 12.17 | 6.56 | 166.63 | 232.14 | 188.87 | 13.03 | 45.54 | 65.72 | 25.02 | 26.55 | 4.05 | 0.40 | 15.92 | 0.70 |
| Uzbekistan | 3.33 | 1.88 | 54.74 | 220.02 | 265.43 | 5.38 | 47.76 | 2.63 | 30.66 | 30.59 | 1.82 | 0.29 | 7.48 | 0.63 |
| Vanuatu | 1.21 | 0.77 | 21.56 | 205.30 | 473.20 | 2.69 | 51.73 | 85.80 | 22.33 | 37.82 | 2.40 | 0.29 | 7.31 | 0.49 |
| Venezuela (Bolivarian Republic of) | 4.91 | 2.58 | 72.89 | 365.27 | 360.09 | 8.17 | 43.80 | 64.46 | 26.54 | 30.00 | 1.32 | 0.38 | 6.60 | 0.61 |
| Viet Nam | 2.13 | 0.92 | 25.02 | 202.79 | 271.41 | 10.57 | 56.38 | 32.01 | 8.16 | 28.96 | 1.31 | 0.41 | 10.94 | 0.62 |
| Yemen | 1.36 | 0.75 | 20.44 | 348.08 | 241.73 | 0.75 | 72.40 | 81.32 | 10.95 | 22.90 | 6.62 | 0.17 | 12.52 | 0.41 |
| Zambia | 2.30 | 1.75 | 46.52 | 142.61 | 204.04 | 6.10 | 97.20 | 92.48 | 21.21 | 18.69 | 2.03 | 0.33 | 4.36 | 0.51 |
| Zimbabwe | 1.23 | 0.93 | 25.23 | 193.335 | 309.686 | 5.13272 | 94.2482 | 99.9536 | 17.153 | 34.8279 | 1.49857 | 0.20675 | 7.48716 | 0.48 |
| ASIR: age standardized incidence rate; ASMR: age standardized mortality rate; ASDR: age standardized DALY rate; SDI: socio demographic index | | | | | | | | | | | | | | |

| **Table 3.** Summary statistics of kidney cancer risk factors for 1990 and 2019 | | | | | | | | | | |
| --- | --- | --- | --- | --- | --- | --- | --- | --- | --- | --- |
|  | 1990 | | | | | 2019 | | | | |
|  | Mean | SD | P25 | Median | P75 | Mean | SD | P25 | Median | P75 |
| ASIR | 2.88 | 2.39 | 1.13 | 1.95 | 3.95 | 4.37 | 3.25 | 1.73 | 3.28 | 6.24 |
| ASMR | 1.70 | 1.23 | 0.75 | 1.17 | 2.38 | 2.16 | 1.34 | 1.07 | 1.76 | 3.00 |
| ASDR | 46.13 | 31.26 | 22.03 | 33.12 | 63.81 | 54.96 | 31.18 | 30.13 | 46.28 | 74.71 |
| Chronic kidney disease | 180.56 | 63.23 | 130.49 | 172.67 | 221.58 | 253.38 | 97.40 | 183.47 | 238.13 | 296.71 |
| Diabetes mellitus type 2 | 215.29 | 95.41 | 150.47 | 193.36 | 258.49 | 321.91 | 141.72 | 220.96 | 283.36 | 385.30 |
| Alcohol use | 6.74 | 4.78 | 2.87 | 5.74 | 10.29 | 7.92 | 5.21 | 3.87 | 7.21 | 11.53 |
| Diet low in fruit | 56.96 | 21.40 | 43.24 | 56.21 | 70.73 | 52.27 | 20.88 | 37.59 | 50.64 | 66.23 |
| Diet low in vegetable | 63.42 | 30.44 | 36.69 | 72.90 | 91.01 | 54.88 | 30.66 | 30.06 | 60.80 | 81.91 |
| High body mass index | 16.13 | 8.57 | 9.07 | 15.92 | 21.62 | 26.10 | 10.61 | 19.03 | 26.26 | 32.64 |
| High systolic blood pressure | 27.93 | 8.42 | 21.84 | 27.11 | 34.61 | 29.99 | 7.36 | 25.20 | 29.99 | 34.88 |
| Lower physical activity | 3.75 | 2.56 | 1.88 | 3.12 | 4.50 | 4.30 | 2.98 | 2.20 | 3.65 | 5.88 |
| Occupation exposure to trichloroethylene | 0.21 | 0.10 | 0.09 | 0.21 | 0.28 | 0.24 | 0.12 | 0.09 | 0.25 | 0.35 |
| Smoking | 12.49 | 6.33 | 6.85 | 11.72 | 17.62 | 10.16 | 5.23 | 5.52 | 9.99 | 13.93 |
| SDI | 0.48 | 0.19 | 0.31 | 0.50 | 0.65 | 0.64 | 0.17 | 0.51 | 0.67 | 0.79 |
| ASIR: age standardized incidence rate; ASMR: age standardized mortality rate; ASDR: age standardized DALY rate; SDI: socio demographic index, SD: standard deviation, P25: 25th Percentile, P75: 75th Percentile | | | | | | | | | | |

| **Table 4.** The adjusted effect of each risk factor on the age-standardized incidence rate, age-standardized mortality rate and age-standardized DALY rate of kidney cancer in the 1990 and 2019 | | | | | | | | | | | | |
| --- | --- | --- | --- | --- | --- | --- | --- | --- | --- | --- | --- | --- |
|  | 1990 | | | | | | 2019 | | | | | |
|  | ASIR | | ASMR | | ASDR | | ASIR | | ASMR | | ASDR | |
| Risk factor | IRR (95% CI) | P | IRR (95% CI) | P | IRR (95% CI) | P | IRR (95% CI) | P | IRR (95% CI) | P | IRR (95% CI) | P |
| Chronic kidney disease | 1.000 (0.999, 1.003) | 0.143 | 1.000 (0.998, 1.003) | 0.509 | 1.000 (0.999, 1.000) | 0.243 | 1.000 (0.999, 1.002) | 0.111 | 1.000 (0.998, 1.002) | 0.662 | 1.000 (0.999, 1.000) | 0.807 |
| Diabetes mellitus type 2 | 0.999 (0.998, 1.001) | 0.954 | 1.000 (0.998, 1.001) | 0.893 | 1.000 (0.999, 1.000) | 0.152 | 0.999 (0.998, 1.000) | 0.223 | 0.999 (0.998, 1.000) | 0.509 | 0.999 (0.999, 1.000) | 0.002 |
| Alcohol use | 1.101 (1.076, 1.126) | <0.001 | 1.093 (1.062, 1.126) | <0.001 | 1.092 (1.086, 1.098) | <0.001 | 1.080 (1.061, 1.099) | <0.001 | 1.061 (1.035, 1.087) | <0.001 | 1.055 (1.050, 1.060) | <0.001 |
| Diet low in fruit | 1.001 (0.996, 1.007) | 0.561 | 1.000 (0.994, 1.007) | 0.807 | 1.001 (0.999, 1.002) | 0.097 | 0.999 (0.995, 1.004) | 0.951 | 1.000 (0.994, 1.006) | 0.958 | 1.000 (0.999, 1.001 | 0.238 |
| Diet low in vegetable | 0.999 (0.995, 1.007) | 0.792 | 0.999 (0.994, 1.005) | 0.921 | 0.998 (0.997, 0.999) | 0.031 | 0.995 (0.991, 0.999) | 0.013 | 0.996 (0.991, 1.001) | 0.159 | 0.996 (0.995, 0.997) | <0.001 |
| High body mass index | 1.026 (1.012, 1.039) | <0.001 | 1.017 (1.000, 1.035) | 0.04 | 1.016 (1.013, 1.020) | <0.001 | 1.017 (1.007, 1.027) | <0.001 | 1.015 (1.001, 1.029) | 0.031 | 1.016 (1.013, 1.018) | <0.001 |
| High systolic blood pressure | 1.001 (0.989, 1.012) | 0.865 | 1.001 (0.986, 1.016) | 0.861 | 1.001 (0.998, 1.004) | 0.289 | 0.998 (0.988, 1.008) | 0.750 | 1.002 (0.988, 1.017) | 0.719 | 1.004 (1.001, 1.006) | 0.004 |
| Lower physical activity | 1.016 (0.976, 1.058) | 0.423 | 1.019 (0.967, 1.074) | 0.462 | 1.014 (1.004, 1.025) | 0.005 | 1.000 (0.971, 1.031) | 0.961 | 0.991 (0.949, 1.035) | 0.705 | 0.987 (0.978, 0.996) | 0.005 |
| Occupation exposure to trichloroethylene | 2.158 (0.671, 6.939) | 0.197 | 2.694 (0.614, 11.810) | 0.189 | 3.516 (2.659, 4.647) | <0.001 | 0.907 (0.402, 2.045) | 0.814 | 1.188 (0.381, 3.701) | 0.766 | 1.548 (1.238, 1.935) | <0.001 |
| Smoking | 1.018 (0.996, 1.040) | 0.094 | 1.011 (0.983, 1.040) | 0.414 | 1.005 (0.999, 1.010) | 0.051 | 1.018 (0.997, 1.040) | 0.088 | 1.015 (0.985, 1.046) | 0.310 | 1.012 (1.006, 1.018) | <0.001 |
| ASIR: age standardized incidence rate; ASMR: age standardized mortality rate; ASDR: age standardized DALY rate; IRR: incidence rate ratio; CI: confidence interval; P: p-value | | | | | | | | | | | | |

| **Table 5.** Detailed multivariate decomposition analysis without socio-demographic index | | | | | | | | | |
| --- | --- | --- | --- | --- | --- | --- | --- | --- | --- |
|  | ASIR |  |  | ASMR |  |  | ASDR |  |  |
|  | Coefficient (95% CI) | P | Percentage | Coefficient (95% CI) | P | Percentage | Coefficient (95% CI) | P | Percentage |
| Endowments | 1.045 (0.602,1.488) | <0.001 | 70.34 | 0.401 (0.085, 0.717) | 0.013 | 87.34 | 10.089 (8.520, 11.658) | <0.001 | 114.23 |
| Coefficients | 0.440 (-0.101, 0.982) | 0.111 | 29.66 | 0.058 (-0.338, 0.455) | 0.774 | 12.66 | -1.256 (-3.253, 0.740) | 0.217 | -14.23 |
| Gap | 1.486 (1.116, 1.855) | <0.001 |  | 0.459 (0.189, 0.728) | 0.001 |  | 8.832 (7.453, 10.212) | <0.001 |  |
| Due to difference in characteristics | | | | | | | | | |
| Risk factor |  |  |  |  |  |  |  |  |  |
| Chronic kidney disease | 0.263 (-0.050, 0.577) | 0.100 | 17.75 | 0.050 (-0.173, 0.275) | 0.658 | 11.05 | 0.142 (-0.999, 1.284) | 0.807 | 1.62 |
| Diabetes mellitus type 2 | -0.177 (-0.464, 0.109) | 0.226 | -11.94 | -0.068 (-0.271, 0.134) | 0.511 | -14.82 | -1.639 (-2.664, -0.614) | 0.002 | -18.56 |
| Alcohol use | 0.340 (0.263, 0.416) | <0.001 | 22.90 | 0.133 (0.078, 0.188) | <0.001 | 28.95 | 3.069 (2.794, 3.344) | <0.001 | 34.75 |
| Diet low in fruit | 0.002 (-0.074, 0.079) | 0.951 | 0.16 | -0.001 (-0.055, 0.052) | 0.958 | -0.32 | -0.162 (-0.432, 0.107) | 0.238 | -1.84 |
| Diet low in vegetable | 0.145 (0.027, 0.262) | 0.015 | 9.78 | 0.058 (-0.024, 0.140) | 0.167 | 12.63 | 1.559 (1.149, 1.970) | <0.001 | 17.66 |
| High body mass index | 0.656 (0.278, 1.033) | 0.001 | 44.16 | 0.283 (0.014, 0.552) | 0.039 | 61.67 | 7.711 (6.355, 9.067) | <0.001 | 87.30 |
| High systolic blood pressure | -0.012 (-0.091, 0.066) | 0.753 | -0.85 | 0.010 (-0.044, 0.064) | 0.713 | 2.21 | 0.411 (0.139, 0.683) | 0.003 | 4.66 |
| Lower physical activity | 0.001 (-0.060, 0.063) | 0.961 | 0.10 | -0.008 (-0.054, 0.036) | 0.706 | -1.91 | -0.333 (-0.569, -0.100) | 0.005 | -3.77 |
| Occupation exposure to trichloroethylene | -0.013 (-0.125,0.098) | 0.816 | -0.90 | 0.011 (-0.065, 0.089) | 0.764 | 2.59 | 0.772 (0.387, 1.158) | <0.001 | 8.75 |
| Smoking | -0.160 (-0.340, 0.018) | 0.079 | -10.83 | -0.067 (-0.194, 0.058) | 0.295 | -14.73 | -1.443 (-2.088, -0.797) | <0.001 | -16.34 |
| Due to difference in coefficients | | | | | | | | | |
| Chronic kidney disease | -0.190 (-1.366, 0.984) | 0.750 | -12.84 | -0.087 (-0.740, 0.566) | 0.794 | -18.94 | -4.199 (-19.616, 11.217) | 0.593 | -47.54 |
| Diabetes mellitus type 2 | -0.275 (-1.180, 0.629) | 0.551 | -18.51 | -0.113 (-0.689, 0.463) | 0.701 | -24.61 | -11.684 (-42.202, 18.833) | 0.453 | -132.28 |
| Alcohol use | -0.399 (-0.931, 0.131) | 0.140 | -26.91 | -0.243 (-0.923, 0.436) | 0.483 | -52.98 | -24.365 (-86.476, 37.745) | 0.442 | -275.85 |
| Diet low in fruit | -0.307 (-1.553, 0.938) | 0.629 | -20.69 | -0.046 (-0.686, 0.593) | 0.887 | -10.12 | -2.132 (-13.934, 9.669) | 0.723 | -24.14 |
| Diet low in vegetable | -0.783 (-1.943, 0.375) | 0.185 | -52.75 | -0.252 (-1.047, 0.541) | 0.533 | -55.05 | -17.417 (-59.081, 24.245) | 0.413 | -197.19 |
| High body mass index | -0.406 (-1.217, 0.404) | 0.326 | -27.33 | -0.052 (-0.502, 0.398) | 0.820 | -11.35 | -1.193 (-9.432, 7.045) | 0.776 | -13.51 |
| High systolic blood pressure | -0.228 (-1.540, 1.083) | 0.732 | -15.40 | 0.042 (-0.676, 0.761) | 0.907 | 9.32 | 7.530 (-12.725, 27.786) | 0.466 | 85.25 |
| Lower physical activity | -0.182 (-0.768, 0.403) | 0.541 | -12.30 | -0.126 (-0.541, 0.288) | 0.550 | -27.57 | -10.628 (-36.516,15.259) | 0.421 | -120.33 |
| Occupation exposure to trichloroethylene | -0.558 (-1.455, 0.337) | 0.222 | -37.58 | -0.204 (-0.842, 0.434) | 0.531 | -44.44 | -17.711 (-62.517, 27.095) | 0.438 | -200.51 |
| Smoking | 0.003 (-1.169, 1.175) | 0.996 | 0.21 | 0.057 (-0.641, 0.756) | 0.872 | 12.51 | 9.829 (-18.210, 37.868) | 0.492 | 111.28 |
| ASIR: age standardized incidence rate; ASMR: age standardized mortality rate; ASDR: age standardized DALY rate; CI: confidence interval; P: p-value | | | | | | | | | |

| **Table 6.** Detailed multivariate decomposition analysis with socio-demographic index | | | | | | | | | |
| --- | --- | --- | --- | --- | --- | --- | --- | --- | --- |
|  | ASIR | | | ASMR | | | ASDR | | |
|  | Coefficient (95% CI) | P | Percentage | Coefficient (95% CI) | P | Percentage | Coefficient (95% CI) | P | Percentage |
| Endowments | 1.426 (0.993, 1.859) | <0.001 | 96.00 | 0.530 (0.210, 0.850) | 0.001 | 115.53 | 12.067 (10.430, 13.705) | <0.001 | 136.63 |
| Coefficients | 0.059 (-0.460, 0.579) | 0.822 | 4.00 | -0.071 (-0.465, 0.322) | 0.723 | -15.53 | -3.235 (-5.267,-1.202) | 0.002 | -36.63 |
| Gap | 1.486 (1.116, 1.855) | <0.001 |  | 0.459 (0.189, 0.728) | 0.001 |  | 8.832 (7.453, 10.212) | <0.001 |  |
| Due to difference in characteristics | | | | | | | | | |
| Risk factor |  |  |  |  |  |  |  |  |  |
| Chronic kidney disease | 0.078 (-0.199, 0.355) | 0.580 | 5.27 | -0.0009 (-0.210, 0.208) | 0.993 | -0.20 | -0.576 (-1.680, 0.526) | 0.305 | -6.53 |
| Diabetes mellitus type 2 | -0.177 (-0.414, 0.059) | 0.142 | -11.95 | -0.073 (-0.254, 0.106) | 0.424 | -16.04 | -1.745 (-2.703, -0.787) | <0.001 | -19.76 |
| Alcohol use | 0.146 (0.056, 0.236) | 0.001 | 9.84 | 0.073 (0.004, 0.142) | 0.036 | 16.03 | 2.174 (1.802, 2.546) | <0.001 | 24.62 |
| Diet low in fruit | -0.014 (-0.080, 0.050) | 0.660 | -0.99 | -0.005 (-0.054, 0.043) | 0.824 | -1.20 | -0.210 (-0.465, 0.044) | 0.105 | -2.38 |
| Diet low in vegetable | 0.068 (-0.031, 0.168) | 0.179 | 4.61 | 0.033 (-0.042, 0.109) | 0.390 | 7.24 | 1.167 (0.765, 1.569) | <0.001 | 13.22 |
| High body mass index | 0.258 (-0.034, 0.605) | 0.080 | 19.21 | 0.160 (-0.091, 0.412) | 0.212 | 34.89 | 5.720 (4.347, 7.094) | <0.001 | 64.77 |
| High systolic blood pressure | 0.009 (-0.055, 0.074) | 0.774 | 0.64 | 0.014 (-0.033, 0.063) | 0.545 | 3.26 | 0.471 (0.215, 0.726) | <0.001 | 5.33 |
| Lower physical activity | -0.027 (-0.081, 0.026) | 0.311 | -1.88 | -0.017 (-0.059, 0.024) | 0.421 | -3.75 | -0.458 (-0.682, -0.234) | <0.001 | -5.19 |
| Occupation exposure to trichloroethylene | 0.030 (-0.060, 0.122) | 0.511 | 2.06 | 0.021 (-0.047, 0.092) | 0.541 | 4.67 | 0.867 (0.507, 1.228) | <0.001 | 9.83 |
| Smoking | -0.121 (-0.270, 0.027) | 0.110 | -8.18 | -0.051 (-0.165, 0.062) | 0.379 | -11.15 | -1.170 (-1.780, -0.559) | <0.001 | -13.25 |
| SDI | 1.149 (0.740, 1.559) | <0.001 | 77.37 | 0.375 (0.051, 0.699) | 0.023 | 81.77 | 5.827 (4.041, 7.613) | <0.001 | 65.97 |
| Due to difference in coefficients | | | | | | | | | |
| Chronic kidney disease | -0.019 (-0.808, 0.770) | 0.962 | -1.30 | 0.019 (-0.838, 0.876) | 0.965 | 4.16 | 2.442 (-3.076, 7.961) | 0.386 | 27.65 |
| Diabetes mellitus type 2 | 0.204 (-2.965, 3.374) | 0.899 | 13.76 | -0.160 (-0.856, 0.535) | 0.651 | -34.93 | -5.976 (-10.373, -1.578) | 0.008 | -67.66 |
| Alcohol use | 0.143 (-2.071, 2.358) | 0.899 | 9.63 | -0.178 (-0.860, 0.504) | 0.609 | -38.78 | -6.538 (-10.738, -2.338) | 0.002 | -74.02 |
| Diet low in fruit | -0.130 (-2.188, 1.927) | 0.901 | -8.77 | 0.127 (-0.760, 1.014) | 0.779 | 27.68 | 3.595 (-1.791, 8.983) | 0.191 | 40.71 |
| Diet low in vegetable | 0.363 (-5.008, 5.735) | 0.894 | 24.46 | -0.317 (-1.401, 0.766) | 0.566 | -69.08 | -9.556 (-15.220, -3.892) | 0.001 | -108.19 |
| High body mass index | -0.080 (-1.365, 1.204) | 0.902 | -5.44 | 0.189 (-0.581, 0.960) | 0.631 | 41.18 | 7.485 (2.801, 12.169) | 0.002 | 84.75 |
| High systolic blood pressure | -0.049 (-1.097, 0.998) | 0.926 | -3.33 | 0.153 (-0.751, 1.058) | 0.740 | 33.30 | 5.749 (-0.020, 11.518) | 0.051 | 65.09 |
| Lower physical activity | 0.173 (-2.373, 2.719) | 0.894 | 11.65 | -0.200 (-0.808,0.408) | 0.519 | -43.57 | -5.812 (-8.930, -2.694) | <0.001 | -65.80 |
| Occupation exposure to trichloroethylene | 0.214 (-3.050, 3.478) | 0.898 | 14.41 | -0.211 (-1.090,0.667) | 0.638 | -45.95 | -7.641 (-12.754, -2.529) | 0.003 | -86.51 |
| Smoking | -0.333 (-5.595, 4.928) | 0.901 | -22.42 | 0.302 (-0.643, 1.248) | 0.531 | 65.81 | 11.285 (4.780, 17.789) | 0.001 | 127.76 |
| SDI | 0.182 (-3.131, 3.496) | 0.914 | 12.28 | -0.485 (-2.655, 1.683) | 0.661 | -105.75 | -21.477 (-35.381, -7.574) | 0.002 | -243.16 |
| ASIR: age standardized incidence rate; ASMR: age standardized mortality rate; ASDR: age standardized DALY rate; CI: confidence interval; P: p-value | | | | | | | | | |
